# Immunogenicity of Recombinant Zoster Vaccine: A Systematic Review, Meta-Analysis, and Meta-Regression

**DOI:** 10.3390/vaccines12050527

**Published:** 2024-05-11

**Authors:** Lorenzo Losa, Ippazio Cosimo Antonazzo, Giuseppe Di Martino, Giampiero Mazzaglia, Silvio Tafuri, Lorenzo Giovanni Mantovani, Pietro Ferrara

**Affiliations:** 1 Center for Public Health Research, University of Milan–Bicocca, 20900 Monza, Italy; 2 Laboratory of Public Health, IRCCS Istituto Auxologico Italiano, 20149 Milan, Italy; 3 Department of Medicine and Ageing Sciences, “G. d’Annunzio” University of Chieti-Pescara, 66100 Chieti, Italy; 4 Unit of Hygiene, Epidemiology and Public Health, Local Health Authority of Pescara, 65100 Pescara, Italy; 5 Interdisciplinary Department of Medicine, Aldo Moro University of Bari, 70121 Bari, Italy

**Keywords:** cell-mediated immunity, herpes zoster, humoral immunity, immunogenicity, recombinant zoster vaccine, vaccine response

## Abstract

Background: The adjuvanted recombinant zoster vaccine (RZV), consisting of varicella-zoster virus glycoprotein E (gE) and the AS01_B_ adjuvant system, effectively prevents herpes zoster (HZ). In the absence of a well-defined correlate of protection, it is important to monitor the RZV immune response, as a proxy of clinical effectiveness. Methods: This systematic review examined post-vaccination parameters: humoral and cell-mediated immunity, avidity index, geometric mean concentration of antibody (GMC), and immunity persistence. The meta-analysis used a random-effects model, and subgroup and meta-regression analyses were conducted. Results: Among 37 included articles, after one month from RZV-dose 2, the pooled response rate for anti-gE humoral immunity was 95.2% (95%CI 91.9–97.2), dropping to 77.6% (95%CI 64.7–86.8) during immunosuppression. The anti-gE cell-mediated immunity-specific response reached 84.6% (95%CI 75.2–90.9). Varying factors, such as age, sex, coadministration with other vaccines, prior HZ, or live-attenuated zoster vaccine, did not significantly affect response rates. RZV induced a substantial increase in gE avidity. Immunity persistence was confirmed, with more rapid waning in the very elderly. Conclusions: This systematic review indicates that RZV elicits robust immunogenicity and overcomes immunocompromising conditions. The findings underscore the need for further research, particularly on long-term immunity, and have the potential to support HZ vaccination policies and programs.

## 1. Introduction

Herpes zoster (HZ), also known as shingles, is an infectious disease that causes painful, unilateral, and vesicular rash. It is caused by the reactivation of latent varicella zoster virus (VZV) and its spread from a dorsal root or cranial nerve ganglion to the corresponding dermatome [1,2]. Individuals who have experienced a primary VZV infection are at risk of developing HZ, and it has been estimated that approximately one third of people may experience a shingles episode during their lifetime [3]. HZ can also recur and cause complications, with postherpetic neuralgia (PHN) being the most common one [3].

Increasing age (≥50 years) and immunosuppressive conditions—such as immunodeficiency or immunosuppression due to disease or therapy—are major risk factors for HZ [4,5]. These conditions are responsible for a decline in cell-mediated immunity (CMI), which is critical in the control of VZV replication and in preventing the reactivation of latent VZV [6,7]. The antibody response also plays additional roles in countering the infection through mechanisms such as preventing cell-to-cell spread and activating natural killer cells to eliminate infected cells [8,9].

Two vaccines are available for the prevention of HZ, namely a live-attenuated zoster vaccine (ZVL, authorized in the United Stated [US] and the European Union [EU] in 2006) and a newer two-dose adjuvanted recombinant glycoprotein E (gE) subunit vaccine (RZV, Shingrix^®^), available since 2017 [10]. The VZV gE is the most abundant glycoprotein in the virus envelope, where it is indispensable for virus replication and cell-to-cell transmission. It also represents a major target for VZV-specific CD4^+^ T-cell immune responses [6,11].

The RZV consists of 50 μg of recombinant VZV gE and the liposome-based AS01_B_ adjuvant system (containing 50 μg of 3-O-desacyl-4′-monophosphoryl lipid A and 50 μg of *Quillaja saponaria* Molina, fraction 21 [QS21]). This is able to promote strong CD4^+^ T-cell and humoral immune responses against recombinant proteins [12,13].

In the pivotal healthy-subject clinical trials, RZV exhibited a vaccine effectiveness of 97.2% in ≥50-year-olds and 89.8% in ≥70-year-old adults [14,15]. Furthermore, the RZV was shown to be effective in reducing the incidence of HZ in frail individuals and patients with immunocompromised conditions [5,16], while long-term studies have confirmed that the protection provided by RZV remains high up to 10 years post-vaccination [17,18].

In the absence of a well-defined correlate of protection [5], monitoring the immune response to the RZV becomes crucial to understand the determinants and predictors of response and to estimate the persistence of the vaccine effectiveness. Research has identified factors influencing varied responses in humoral and cell-mediated immunity and their persistence. For instance, age, with inconclusive findings in some studies [18,19,20,21], and immunosuppressive conditions, like hematological malignancies and transplant recipients, may also impact RZV immunogenicity [22,23,24]. It cannot be excluded that these conditions could be also related to a lower effectiveness in real life. Therefore, this systematic review aims to synthesize the currently available evidence on the immune response after RZV administration and to explain potential discrepancies through meta-analysis and meta-regression, specifically focusing on humoral and cell-mediated responses.

## 2. Materials and Methods

### 2.1. Study Design

Our systematic review and meta-analysis of RZV immunogenicity adhered to the PRISMA 2020 guidelines for reporting [25] and followed a predefined study protocol registered in the International Prospective Register of Systematic Reviews (PROSPERO), with the number CRD42023459621 [26]. We considered the following endpoints for post-vaccination immunogenicity: humoral immunity, CMI, avidity index, geometric mean concentration of antibody (GMC), and persistence of immunity.

### 2.2. Search Strategy and Selection Criteria

We conducted a comprehensive literature search from inception up to 31 August 2023, with an update on 9 October 2023, in multiple databases, including PubMed/MEDLINE, Embase, Web of Science, and Global Index Medicus. Supplemental Table S1 details the search strategy. Additionally, we reviewed the reference lists of the full texts we included in our analysis. The results were restricted to articles in English, French, German, Italian, and Spanish. We did not set any time limit on dates of published articles. During the screening process, two reviewers (L.L. and I.C.A.) independently assessed titles, abstracts, and full texts of the studies identified in our searches. Any discrepancies between reviewers regarding title and abstract screening, full-text review, or reasons for exclusion were resolved through group discussion with the principal investigator (P.F.). Populations eligible for this review were anyone aged 18 years or older who received two doses of RZV. Eligible study designs included: (i) primary reports published as randomized controlled trials (RCT), quasi-RCT (qRCT), cohort, cross-sectional, and case–control studies; (ii) original reports accessible in full-text; (iii) studies indicating time between vaccination and testing. Records not reporting immunogenicity data as well as those published as reviews, case report, conference abstract, position paper, editorial, commentary, and letters without original data were excluded. Inclusion/exclusion criteria were developed according to the PICOS (Participants, Interventions, Control, Outcomes, Study Design) framework and detailed in Table S2 (Supplementary Materials). Two reviewers (L.L. and I.C.A.) assessed the possible risk of bias using the Cochrane risk of bias tool and the JBI (formerly Joanna Briggs Institute) checklist for RCTs and non-randomized experimental studies, respectively [27,28].

### 2.3. Data Analysis

We extracted the first author’s last name, country and year of publication, study design, sample size, participants’ age and sex (i.e., the proportion of women; when not available for each subgroup, the proportion of women for the whole sample was used as a proxy estimate for subgroups), history of HZ and previous vaccination with ZVL, immunocompromising illnesses, vaccine co-administration, assay and cut-off value employed to measure the antibody level and CMI, time between RZV doses, time between RZV-dose 2 and blood sampling, and description of the endpoints of interests. Many studies in our search presented results for vaccination across a range of ages, and for consistency we used the lower limit of the range as our reference point. If the exact number for an immunogenicity endpoint was not clearly reported in the study text, we calculated it from the related percentage. Only per-protocol results were derived from RCTs.

Whenever feasible, we provided separate data for subgroups within studies. We computed random effects meta-analyses to estimate the pooled proportions (i.e., the vaccine response rate [VRR]) for anti-gE humoral and CMI immune responses using the logit transformation for standard error. Results were expressed as pooled VRR with a 95% confidence interval (95%CI). Heterogeneity between the results of different studies was measured with the *I*^2^ statistic [29].

For humoral VRR, we only included reports that measured anti-gE antibody concentrations through an enzyme-linked immunosorbent assay (ELISA) with a cut-off for seropositivity of 97 milli-International Units (mIU)/mL. Humoral response was defined as the proportion of participants with a fourfold or greater increase in anti-gE concentration post-RZV-dose 2, as compared to pre-vaccination for initially seropositive participants or compared to the antibody cut-off value (97 mIU/mL) for participants who were seronegative at pre-vaccination [18].

For our assessment of anti-gE GMC, we only reviewed reports with within-study comparisons of RZV-vaccinated participants, thus excluding comparisons with placebo. We analyzed the GMC ratio (GMC_RZValone_/GMC_CoAd_) and 95%CI for vaccine co-administration through random-effects meta-analysis, including GMC values calculated conditionally to the means of the log-transformed concentrations. The non-inferiority threshold was determined as the point at which the upper limit of the 95%CI for GMC ratio was less than 1.5.

To describe antibody avidity, we sought studies that investigated the effect of adding diethylamine (DEA) to an antibody–antigen mixture, where low avidity antibodies, which have weaker binding to antigens, are more likely to dissociate from the antibody–antigen complexes than those with higher avidity. This allows us to determine the avidity expressed as avidity index (AI), calculated as the result of optical density (OD) from plates washed four times with DEA, divided by the OD from plates used for conventional gE ELISA, and multiplied by 100 (high values indicate highly avid antibodies) [30].

We assessed the gE-specific CMI responses VRR as the frequency of CD4^+^ T cells, measured through flow cytometry, expressing two or more of the following activation markers (double-positive CD4^+^ T cells, hereafter termed CD4^2+^ T cells): CD40 ligand (CD40L), interferon-γ (IFN-γ), interleukin-2 (IL-2), and tumor necrosis factor-α (TNF-α) [31]. VRR was defined as the percentage of participants with post-vaccination CD4^2+^ T cell frequencies (i) ≥2-fold the cut-off (320 positive cells per 10^6^ CD4^+^ T-cells counted) for participants initially below the cut-off or (ii) ≥2-fold the pre-vaccination CD4^2+^ T-cell frequencies for participants initially above the cut-off.

For immunogenicity endpoints, we conducted a meta-analysis using data obtained from the examination of blood samples obtained one month after RZV-dose 2, which corresponds to the peak immune response [18], as well as longer-term intervals where available. We fitted subgroup and random-effects meta-regression analyses to examine heterogeneity for endpoints with at least ten comparisons [29,32]. Moderating variables were initially assessed separately in univariable meta-regression models before being examined together in a single model [32]. If two or more variables were found to be significant in the univariable analysis, a multivariable model was constructed for each outcome. Univariable random-effects meta-regressions included the following mediators: age at vaccination and sex, immunocompromising illnesses, history of HZ, previous vaccination with ZVL, vaccine co-administration, study design, and area of study. For comparisons with at least 10 reports, the graphical evaluation of the funnel plots and the Egger’s regression asymmetry test (with statistical threshold at *p* < 0.10) were used to compute potential publication bias [33].

We used meta and metafor packages in R 4.2.1 statistical software for the analysis [32,34,35,36]. For studies that did not meet criteria for inclusion in the meta-analysis, we reported the results in a narrative synthesis.

## 3. Results

### 3.1. Literature Search

**Figure 1:**
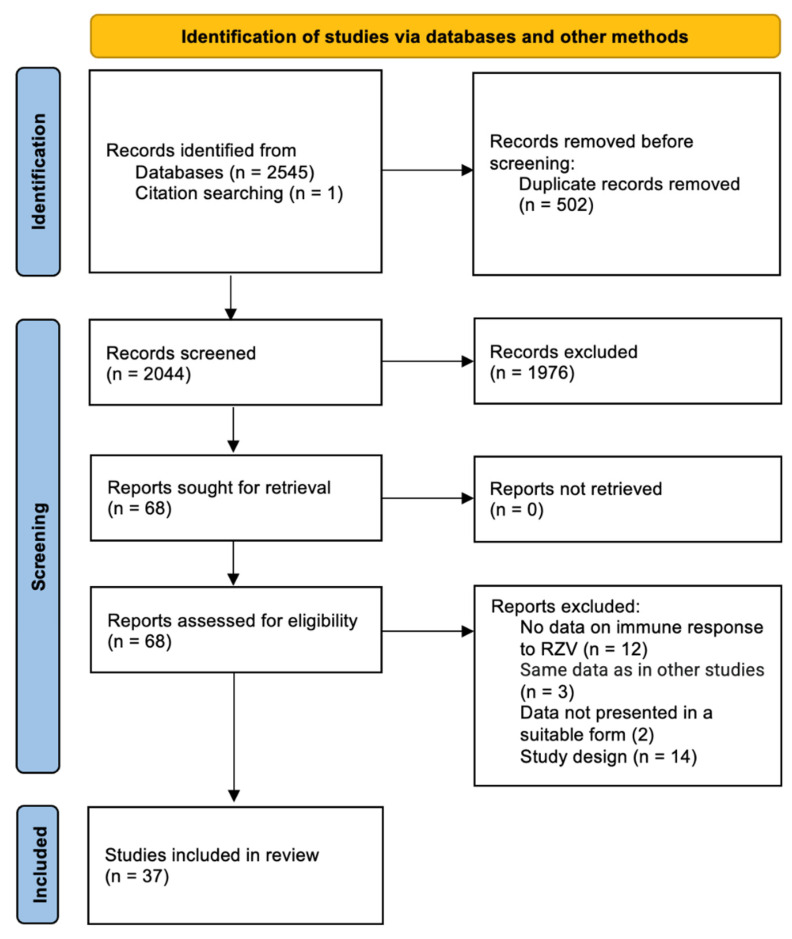
PRISMA flow diagram of the study selection process.

Our initial search yielded 2546 records. After the exclusion of duplicates and the screening of titles and abstracts, 68 articles were assessed for eligibility via full-text evaluation (Figure 1).

Thirty-seven studies in total meet the inclusion criteria and were included in the systematic review (see Supplementary S1 for the full list of included articles and Table S3 for the full texts excluded with reasons, Supplementary Materials). These were published from 2012 to 2023, 31 as RCTs, 2 as qRCTs, and 4 as cohort studies. Overall, we extracted 84 different reports, each corresponding to a unique sub-cohort, which are presented in Table 1. Fifty-four reports enrolled participants in good health, except for those affected by chronic non-communicable diseases that do not reduce the immune capacity (hereafter defined as immunocompetent adults), while the remaining reports included participants with primary or secondary immunosuppression in patients with solid (2 sub-cohorts) or hematological malignancies (10), solid organ transplant (SOT) recipients (6), hemopoietic stem cell transplantation (HSCT) recipients (11), and people living with HIV (1). Regarding the immunological endpoints, 75 records provided pertinent information on humoral immunity, 47 on CMI, 4 on avidity, and 27 on within-study GMC comparisons. Definitions of endpoints not accepted for inclusion in the meta-analysis are detailed in Table S4 (Supplementary Materials).

### 3.2. Humoral Immunity

**Figure 2:**
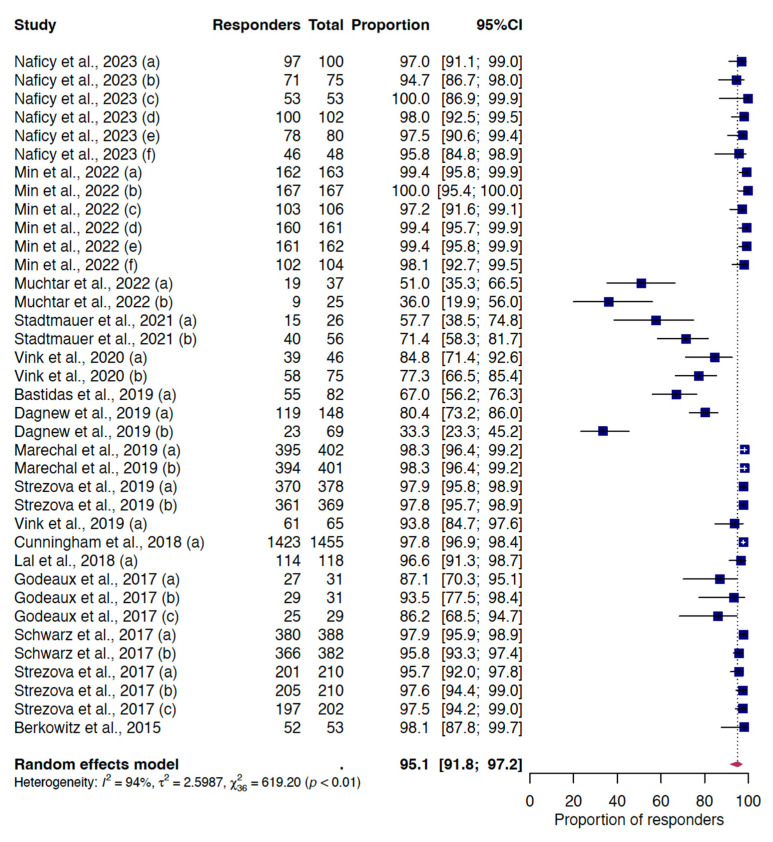
Random-effect meta-analysis of the VRR for humoral immunity one month following RZV-dose 2. Letters in brackets indicate the different reports extracted from individual studies included. For the complete list of included studies, please refer to the Supplementary Materials, Supplementary S1. Abbreviations: VRR, vaccine response rate; RZV, recombinant zoster vaccine; 95%CI, 95% confidence interval.

We estimated a pooled VRR for anti-gE humoral immunogenicity of 95.2% (95%CI 91.9–97.2), derived from the analysis of 37 reports that provided data after one month following RZV-dose 2 in 6609 participants (Figure 2). The pooled estimates for subgroup and meta-regression analyses are presented in the Supplementary S2 (Tables S5 and S6, Supplementary Materials). In the group of individuals with immunocompromising conditions (which also included the youngest vaccinated), the VRR dropped to 77.6% (95%CI 64.7–86.8), while in immunocompetent recipients it settled at 97.8% (95%CI 97.3–98.2), while the pooled proportion of RZV recipients with positive humoral response reached 95.2% (95%CI 91.9–97.2). This finding was consistent with multivariable meta-regression results (Table S6, Supplementary Materials). Subgroup analysis also showed a difference depending on the dosing interval, but this difference disappeared when considering only individuals with immunosuppressed conditions. No differences were observed in cases of co-administration with other vaccines or the inclusion of individuals with a previous history of HZ. With respect to potential determinants of antibody response in studies not included in the meta-analysis, few studies have described differences related to selected characteristics.

There were no significant differences in anti-gE antibody response one month following RZV-dose 2 as a function of age in six studies [20,23,24,37,38,39], sex in four [20,23,37,38], prior ZVL vaccination in one [20], or the time interval from lung transplant to vaccination in one [24]. Godeaux et al. described the lowest VRR in vaccinated people with the most recent HZ episode history (≤4 years vs. >4 years) [40]. Zent et al. reported a significant correlation between a longer duration of Bruton tyrosine kinase inhibitor (BTKi) therapy and poorer post-vaccination anti-gE concentration in patients with chronic lymphocytic leukemia/small lymphocytic lymphoma (CLL) and lymphoplasmacytic lymphoma (LPL) [38].

### 3.3. Geometric Mean Concentration

**Figure 3:**
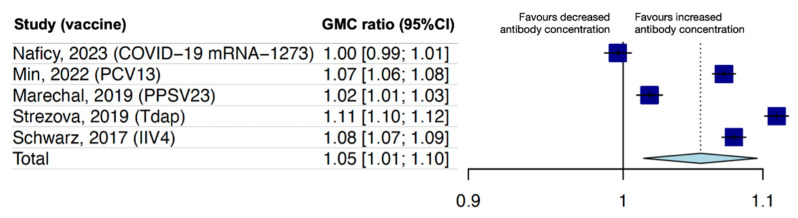
Random-effect meta-analysis of within-study comparisons of GMCs following the co-administration of RZV with other routine vaccines. In all studies, routine vaccines were co-administered with RZV-dose 1, and the measurement of anti-gE humoral immunity was conducted one month after RZV-dose 2. Noninferiority of the anti-gE antibody response was demonstrated if the upper limit of the 95%CI of the GMC ratio (RZV alone over co-administration) was <1.5, one month after RZV-dose 2. Abbreviations: RZV, recombinant zoster vaccine; GMC, geometric mean concentration of antibody; 95%CI, 95% confidence interval; COVID-19, coronavirus disease 2019; PCV13, 13-valent pneumococcal conjugate vaccine; PPSV23, 23-valent pneumococcal polysaccharide vaccine; Tdap, tetanus–diphtheria–pertussis vaccine; IIV4, quadrivalent seasonal inactivated influenza vaccine.

In eight studies with within-study comparisons of GMC, five evaluated co-administration of the first dose of RZV with other routine adult vaccines, such as the COVID-19 mRNA-1273 vaccine, the 13-valent pneumococcal conjugate vaccine (PCV13), the 23-valent pneumococcal polysaccharide vaccine (PPSV23), the tetanus–diphtheria–pertussis vaccine (Tdap), or the quadrivalent seasonal inactivated influenza vaccine (IIV4) [41,42,43,44,45]. The overall pooled GMC ratio for individuals vaccinated with RZV alone compared with vaccine co-administration was 1.05 (95%CI 1.01–1.10), demonstrating noninferiority between groups (Figure 3).

Lal et al. found that, compared with the standard 0–2-month schedule, the GMC non-inferiority criterion was met for RZV doses administered 6 months apart, but not for the 0–12-month schedule [46]. In Godeaux et al., the post-vaccination anti-gE GMCs were comparable for all ≥50-year age groups and between study participants with different timeframes since the previous HZ episode [40]. Grupping et al. observed that the humoral response to RZV was noninferior in adults previously vaccinated with ZVL when compared with ZVL-naïve recipients [47]. In Vink (2017) et al., the subcutaneous administration of RZV was noninferior to the intramuscular in eliciting the antibody response [48].

### 3.4. Antibody Avidity

Data on post-RZV antibody avidity were derived from four studies, including a total of 304 participants. In Weinberg (2023) et al., the peak response of 95.5 (8.6 SD) AI corresponded to 30 days after the second dose, without differences based on sex, age, or prior ZVL administration [20]. In the study by Schmid et al., 48% of the recipients exhibited avidity ≥50 AI and 23% ≥60 AI, without differences of age or prior ZVL administration [30]. Two studies enrolled SOT recipients: the median value of antibody avidity increased from 0% (IQR 0–0) before vaccination to 12% (IQR 0–60) one month after RZV in L’Huillier et al. [49], whereas AI reached 84.2% (IQR 59.4–96.5) in lung transplant recipients at the 3–6-week assessment in Hirzel et al.’s study [24].

### 3.5. Cell-Mediated Immunity

**Figure 4:**
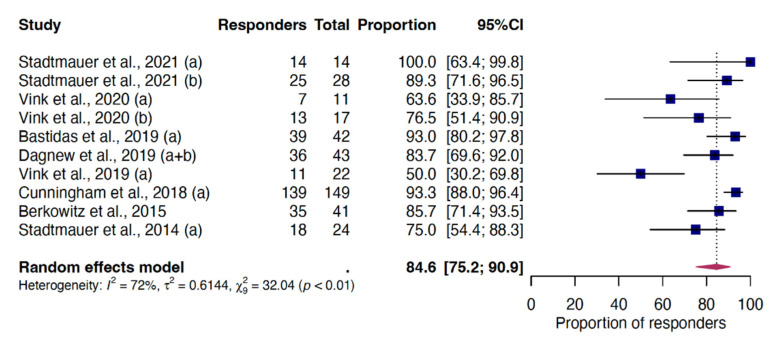
Random-effect meta-analysis of the VRR for cell-mediated immunity one month following RZV-dose 2. Letters in brackets indicate the different reports extracted from individual studies included. For the complete list of included studies, please refer to the Supplementary Materials, Supplementary S1. Abbreviations: VRR, vaccine response rate; RZV, recombinant zoster vaccine; 95%CI, 95% confidence interval.

The analysis of the CMI VRR was calculated on 391 recipients enrolled in 10 reports, resulting in a pooled proportion of 84.6% (95%CI 75.2–90.9) one month post-dose-2 (Figure 4). The results of the subgroup and meta-regression analyses (Tables S7 and S8, Supplementary Materials) show a reduced response when the two doses are administered less than 2 months apart, with a 19.4% (95%CI 5.8–40.0) reduction in the response rate between administrations at one and one/two months vs. two months. Excluding the single report that included only healthy individuals [18], this reduction remained at 16.0% (95%CI 3.3–35.7). A post hoc analysis of median CD4^2+^ frequency values extracted from the studies included in the CMI meta-analytic model, though, did not reveal any differences based on the time between the two doses (Figure S1, Supplementary Materials).

Previous vaccination with ZVL did not impact the gE-specific CD4^2+^ clone response, as seen in five studies [20,37,47,50,51]. No effects related to the participants’ sex or age on CMI response were observed, respectively, in four [23,37,50,51] and six [24,37,39,50,51,52] studies. The CD4^2+^ T-cell frequencies in SOT recipients were in similar ranges to those observed in immunocompetent adults ≥50 years of age [24,53]; this level appeared to be higher the longer the interval between transplantation and vaccination in lung transplant recipients [24]. L’Huillier et al. observed that lung transplant recipients had lower median anti-gE CD4^2+^ T-cell counts than other organ transplant recipients [49].

Delving deeper into the CD4^2+^ T cells’ activation markers after RZV, a difference in their frequency can be described. Five studies observed that cells expressing CD40L or IL-2 had a higher frequency than those expressing IFN-γ or TNF-α [18,19,24,49,54,55].

### 3.6. Persistence of Immunity

**Figure 5:**
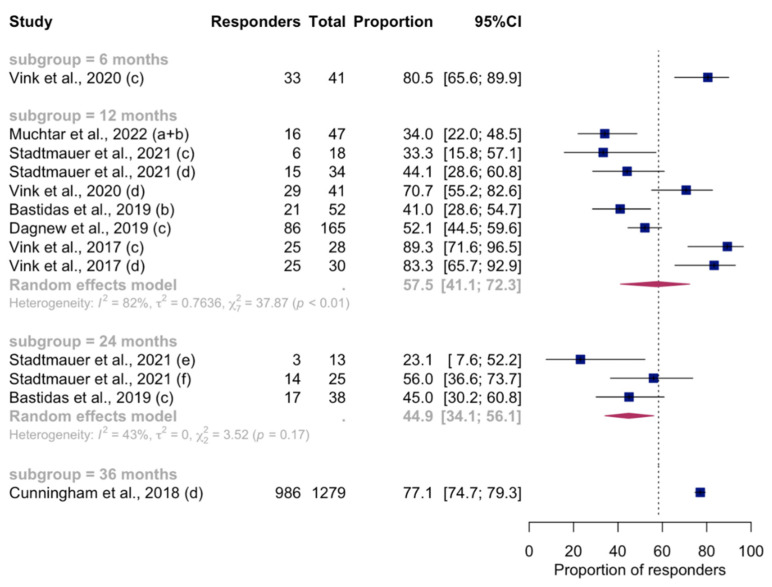
Time-varying pooled VRRs (with 95%CI) of the proportion of RZV recipients with positive humoral immunity response, by time since vaccination following RZV-dose 2. Only studies meeting criteria for inclusion in the meta-analysis were pooled. Letters in brackets indicate the different reports extracted from individual studies included. For the complete list of included studies, please refer to the Supplementary Materials, Supplementary S1. Abbreviations: VRR, vaccine response rate; RZV, recombinant zoster vaccine; 95%CI, 95% confidence interval.

**Figure 6:**
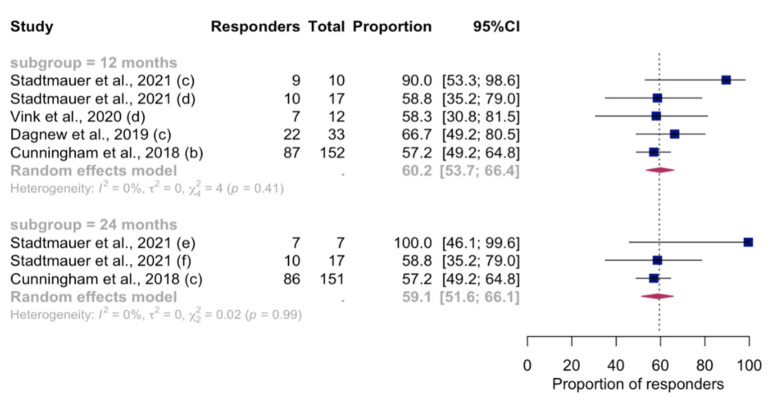
Time-varying pooled VRRs (with 95%CI) of the proportion of RZV recipients with positive cell-mediated immunity response, by time since vaccination following RZV-dose 2. Only studies meeting criteria for inclusion in the meta-analysis were pooled. Letters in brackets indicate the different reports extracted from individual studies included. For the complete list of included studies, please refer to the Supplementary Materials, Supplementary S1. Abbreviations: VRR, vaccine response rate; RZV, recombinant zoster vaccine; 95%CI, 95% confidence interval.

Considering the studies that met inclusion criteria in the meta-analysis, longer longitudinal serological assays from 13 reports revealed a progressive decline in the humoral VRR from 6 to 24 months after vaccination (Figure 5). Figure 6 shows longitudinal analyses of CMI-specific VRR at 12- and 24-month follow-ups, in which the proportion of vaccinated people with a positive cell-mediated response remained at around 60%. For both humoral and CMI long-term VRR, it was not possible to proceed with subgroup analyses or meta-regression because of the low number of reports (<10 for each post-vaccination timepoint). In the remaining part of the studies not included in the synthesis model, the decline in either or both humoral immunity (in terms of GMC/antibody titer) and CMI (expressed as CD4^2+^ T-cell frequency) was confirmed, but in all the studies it was found that the level remained higher than the baseline. These data were confirmed at different timepoints up to 10 years after vaccination [17,20,21,23,50,54,55,56,57,58,59,60].

For humoral response, Weinberg (2023) et al. found that individuals ≥70 years old had lower anti-gE levels at months 24 and 60, compared to those 50–59 years old [20]. In Cunningham et al., VRRs at 12, 24, and 36 months following RZV-dose 2 were slightly lower in recipients aged 70 years and over than in those aged 50–69 years [18]. In HSCT patients, humoral immune responses in the 18–49-year-old group decreased to nearly their baseline levels within 24 months post-vaccination [19]. In two studies, long-term anti-gE concentrations were in similar ranges between 60 and 69- and ≥70-year-old groups, up to 72 and 108 months [21,59].

For CMI response, Cunningham et al. found that, compared to individuals aged under 70 years, a slightly smaller percentage of ≥70-year-old recipients remained above the VRR threshold, and they also tended to have lower CD4^2+^ T-cell frequencies at all timepoints [18]. In patients with solid tumors, CMI VRR was higher in those aged 18–49 years (compared to those ≥50 years old) at all postvaccination timepoints [52]. Two studies reported that the long-term frequencies of gE-specific CD4^2+^ T cells were lower in ≥70-year-old recipients than in those <70 by month 12 [18,59], although they remained substantially higher than baseline levels [18]. No age effect on the kinetics of Th1 responses to gE was observed in two studies [21,50]. Sex or prior ZVL vaccination did not predict the persistence of anti-gE antibodies [20,55], CMI response [50,55], or avidity [20]. The anti-gE avidity remained significantly higher up to 5 years after RZV [20,30], with no difference based on sex [30] or prior ZVL [20,30]. Weinberg (2023) et al. observed that 50–59-year-old recipients had higher anti-gE avidity than adults ≥70 years old at years 2 and 5 [20].

### 3.7. Risk-of-Bias Assessment

Among the 37 studies considered, 7 (consisting of 5 RCTs and 2 cohort studies) exhibited a high risk of bias and 28 studies (comprising 26 RCTs and 2 cohort studies) were characterized as having a low risk of bias. The remaining two RCTs fell into the category of “some concerns”. Notably, issues with randomization and missing outcome data were primary sources of bias in RCTs, while cohort studies lacked a control group (Tables S9 and S10, Supplementary Materials). Additionally, the analysis of publication bias in the model for humoral VRR revealed an asymmetry in the funnel plot, and this was further confirmed through Egger’s linear regression test (Table S11, and Figures S2 and S3, Supplementary Materials). Heterogeneity in the meta-analytic models was roughly moderate to high.

## 4. Discussion

To our knowledge, this is the first systematic review assessing immunological responses to RZV administered in adult populations. Our study indicates that the two-dose RZV is a highly immunogenic vaccine, resulting in strong T-cell immunity and antibody response. Studies have also reported a positive correlation between anti-gE antibody concentrations and gE-specific CD4^2+^ T-cell frequencies [18,20,49,55].

Immunogenicity was also confirmed in individuals with primary or secondary immunodepression, although somewhat less so than in immunocompetent recipients, in line with findings from a previously published review conducted on immunocompromised adults [5]. While the overall pooled proportion of RZV recipients with positive humoral response reached 95.2% (95%CI 91.9–97.2) one month following vaccination, multivariable meta-regression analysis confirmed that the presence of immunocompromising conditions was a predictor of the VRR, decreasing the proportion of individuals who presented a seropositive result when vaccinated with two doses of RZV. The reduction in the gE-specific CMI response in this population appears to be lower or non-existent compared to humoral immunity, but the number of studies included in the meta-analysis is too low to draw robust conclusions.

A specific case arises in patients with hematological malignancies, in which a variable response is observed across primary studies. In various conditions—including CLL, LPL, monoclonal B cell lymphocytosis, and non-Hodgkin B-cell lymphoma (NHBCL)—the immunological parameters post-RZV are reduced compared to the general population, and a more pronounced reduction was observed in NHBCL patients. This decrease in humoral VRR is primarily attributed to the type of treatment administered in these neoplasms. Patients receiving anti-CD20 monoclonal antibodies (i.e., rituximab), B-cell lymphoma 2 inhibitors (i.e., venetoclax), and BTKis were less likely to mount an adequate response [19,23,37,38,61,62]. These findings are consistent with previous research showing that exposure to agents that cause B-cell depletion or the disruption of B-cell receptor signaling diminishes humoral responses to mRNA COVID-19, influenza, pneumococcal polysaccharide, and vaccines [63,64,65]. T-cell irregularities—such as an elevation in T-regulatory cells, exhausted T cells, and difficulties in forming effective immunological synapses—have been described in certain hematological malignancies, further explaining the reduced CMI response following RZV vaccination [37,66]. Reduced humoral response, but not CMI, was also reported in adults who had undergone HSCT, likely due to the high-dose immunosuppression regimens [19,22]. However, the vaccine has shown an efficacy of 87.2% in preventing HZ and PHN among adults with hematological malignancies [61], and of 68.2% in HSCT recipients [22], in line with the role of the CMI response as the primary mechanistic driver of protection against HZ [5,6]. In SOT recipients, the levels of gE-specific humoral GMCs and VRRs, as well as CMI VRRs, appear to be lower than those in immunocompetent adults [49,53]. Interestingly, two studies described an inverse correlation between the use and dose of mycophenolate and vaccine-elicited anti-gE response [24,49], mirroring findings reported previously with the influenza vaccine [67]. Specific attention should be given to lung transplant recipients, as they are one of the most vulnerable groups to HZ among SOT recipients [24,68]. While L’Huillier et al. found no disparity in median anti-gE levels between lung and non-lung transplant recipients one month after RZV-dose 2 [49], and Hirzel et al. showed that AI reached values indicative of a significant antibody response [24], the scenario differs for the CMI. Indeed, the first study also found that lung transplant recipients had lower median double-positive polyfunctional CD4^2+^ T-cell counts than other SOT recipients, which likely correlates with the routine therapy [49]. However, the number of lung transplant recipients enrolled was low, and future research will be needed to further clarify aspects related to the immune response to RZV in this specific population. Finally, in line with the results observed in immunocompetent individuals, Hirzel et al. demonstrated that double-positive CD4^2+^ T cells expressing CD40L and IL-2 had a higher frequency (than those expressing IFN-γ or TNF-α) in this group, suggesting that the immunosuppressive regimen does not appear to significantly impact the profile of polyfunctional gE-specific CD4^2+^ T cells [24].

The clinical evidence shows that CMI response is critical in the protection against HZ [6,7,8]. We observed that the proportion of individuals achieving a positive CMI response is higher when the two doses are administered two months apart compared to intervals of less than two months. Additionally, this analysis remains consistent even when conducted exclusively on non-immunocompetent individuals. However, among those individuals who respond, there is no noticeable difference in CD4^2+^ T-cell frequency. It is important to conduct additional research on this aspect to confirm the potential of a clinical correlation associated with this difference, regardless of the overlap in confidence intervals, which may be due to a lack of information rather than the absence of true difference [29]. This is also influenced by the heterogeneity of the populations included in the meta-analysis. On this point, it is worth mentioning that the US Advisory Committee on Immunization Practices acknowledges that, in clinical practice, administering doses too closely together may result in a suboptimal immune response [69], even though there is a lack of definitive evidence. The issue is also linked to the need for an accelerated RZV schedule to protect the patient when initiating immunosuppressive therapies or in other immunodeficiency disorders. Indeed, determining the optimal timing of vaccination in patients receiving immunomodulatory therapies is a crucial aspect for HZ prevention. Previous research has proposed potential approaches, including booster schedules at specified time intervals or temporary therapy discontinuation for clinically stable patients during vaccination [23]. It is imperative that dedicated research validates the feasibility, assesses the clinical benefits, and evaluates the risks of these strategies. An important recommendation, however, is to administer the vaccine, whenever possible, prior to the initiation of the immunosuppressive treatment or chemotherapy, as well as in the pretransplant period. This, however, represents an advancement compared to the availability of ZVL alone, in which vaccine administration was often discouraged because patients would need to postpone their transplant for four weeks after each live vaccine [49], due to the risk of varicella resulting from the vaccine strain during immunosuppression [70].

Anti-gE CD4^2+^ T-cell polyfunctionality is thought to be one of the main mechanistic immune correlates of protection conferred by RZV [71,72]. CD40L, which is key for the development of CD4^+^ T-cell-dependent effector functions, was the most expressed marker, either alone or in combination with IL-2, considered in turn as a predictor of immunogenicity [72], and followed by combinations with the other markers. This expression pattern mirrors the signature of polyfunctional profiles observed after the administration of the hepatitis B surface antigen with AS01_B_ adjuvant [18,73,74]. This represents a novel aspect in protection against HZ, distinct from the immune response induced by ZVL. Research indicates that AS01_B_ significantly boosted anti-gE T-cell responses, even in the oldest age group and in non-immunocompetent individuals [39,62], through various mechanisms including the activation of macrophages and the production of IFN-γ, with the latter having a crucial role in the immune response against VZV [18,75,76].

Strong gE avidity exhibits higher correlations with VZV neutralization and enhances protection [8,30]. Evidence for this functional quality of the anti-gE antibodies was available from four studies. They suggested that RZV stimulates a significant and long-lasting increase in gE avidity in most recipients, demonstrating superiority over ZVL, for which avidity is less marked and tends to drop after year 1 [20,24,30,49]. The strength with which antibodies bind to the gE is lower during immunosuppression [49].

We found evidence on the persistence of immune response to RZV for up to 10 years following the initial vaccination. In terms of the duration of immunity, research suggests that both humoral and CMI responses have been observed to plateau at approximately four years after vaccination [21,59]. The dynamics of RZV-induced immunity are consistent with the sustained clinical benefits of the vaccine. The long-term follow-ups of the ZOE-50/70 clinical trials reveal that the vaccine efficacy against HZ remained consistently high (>70%) up to 10 years after the initial 2-dose vaccination regimen administered to more than 6000 participants aged 50 years or older [17,60]. The persistence of both humoral and cell-mediated immunities was dependent on age at RZV administration. Several studies have described how the proportion of individuals with a positive response in terms of both anti-gE antibodies and anti-gE CD4^2+^ T cells tends to decrease with advanced age (≥70 years), although these immunological parameters remain substantially higher than baseline levels [17,20,21,23,50,54,55,56,57,58,59,60]. This opens up the discussion on the waning of RZV immunity and understanding whether a booster dose is needed, especially in older and immunocompromised individuals. While predictive analyses exist that model the persistence of immunity for up to 15 years after the initial vaccination [21], additional follow-up is needed to gather further information on the long-term immunogenicity and effectiveness of RZV.

Again, on the persistence of CMI response, the study conducted by Laing et al. discovered that the long-lasting CD4^+^ T cell response induced by RZV represented the vaccine’s ability to recruit naïve CD4^+^ T cells (rather than clonotypes derived from memory pools), and that the frequency of those clones correlates with the frequency of precursors among naïve CD4^+^ T cells prior to vaccination [56].

Before the introduction of RZV, the prevention of HZ relied on ZVL. While comparison with the immunogenicity induced by ZVL was not among the systematic review’s outcomes, in the retrieved literature RZV has been shown to compare favorably in immunological response. In all studies comparing the two vaccines, RZV recipients had a higher gE-specific humoral response rate and levels of anti-gE antibodies, higher CD4^+^ T-cell responses and gE-specific avidity [18,30,50,51,54], as well as longer persistence of immunity—in all timepoints after vaccination up to 60 months [20,30,50]—than ZVL. These findings are consistent with the RZV’s long-term efficacy and with the declining protection of ZVL. Of note, a significant decline in the effectiveness of ZVL was reported, dropping from 68.7% in the first year to 4.2% in the eighth year [77].

In brief, the available evidence suggests that RZV provides robust immunological protection against herpes zoster in older adults and in adults under major immunosuppressive conditions. This vaccine is indeed particularly suitable for individuals affected by primary or secondary immunosuppression and young chronic patients [5]. The results of the work presented here should be considered in relation to the analysis and synthesis of primary research on RZV’s effectiveness in reducing the burden associated with HZ. Lastly, in vaccination strategies for the general older population, there is a need to consider the costs of the two available HZ vaccines and conduct comparative cost-effectiveness analyses [78].

Several methodological issues and limitations to the present study warrant discussion. First, the studies included in this synthesis displayed differences and overlaps in age ranges within their participant cohorts, which could introduce a potential aggregation bias [29,79]. For many subgroups, it was not possible to track the average or median age, as well as the upper age range limit. This variance is also evident in the heterogeneity of the results, and the meta-regression models had challenges dealing with non-independent age ranges, adding complexity to the analysis. This choice of using the lower limit of the ranges, however, was essential to ensure the inclusion in the analysis of one of the essential characteristics in the assessment of vaccine response. Second, it is remarkable that the number of immunocompromised patients per study was low compared to those enrolling immunocompetent participants. Information regarding vaccine immunogenicity in immunocompromised recipients is therefore still limited, and the limited sample sizes within each patient category provided insufficient statistical power for comparisons between conditions’ groups. It is also important to add that the concept of immunocompromisation is very broad and includes, for example, people living with HIV with a normal lymphocyte count and people with confirmed AIDS [80]. Third, this also applies to the analyses of the avidity index and CMI. For the latter, except for Cunningham et al.’s study, which observed these data in 149 participants, the rest of the studies (all on non-immunocompetent individuals) enrolled fewer than 45 patients each, while many studies on CMI response were not included in the meta-analysis because they did not meet the inclusion criteria (Table S4, Supplementary Materials). This limitation strongly advocates for further research on the CMI and avidity responses of RZV. Fourth, for both humoral and CMI long-term VRR, it was not possible to proceed with subgroup analyses or meta-regression for the low number of reports (<10 for each post-vaccination timepoint). Fifth, this meta-analysis evaluates the immune response and potential differences observed in vaccine response that must be considered together with their clinical correlates (i.e., potential differences in effectiveness) in terms of potential medium to long-term clinical outcomes such as episodes of HZ or hospitalizations. Finally, there was moderate to high heterogeneity in some important variables that complicated comparisons across studies.

## 5. Conclusions

In conclusion, the findings of our review suggest that the administration of RZV to adults elicits a robust and long-lasting immune response, overcoming immunosenescence and many immunosuppressive conditions, although somewhat reduced immunity has been revealed in particular groups (i.e., reduced VRRs in the case of immunodepression or a more significant immunity decrease in the very elderly). Further research on vaccine administration during immunomodulatory treatments and in immunocompromised conditions, immunological analyses associated with real-world effectiveness studies, the optimal dosing interval, and the persistence of immunity could help to inform the best RZV vaccination schedules required to achieve coverage among those who are recommended to receive HZ vaccination, primarily older adults and people with primary or secondary immunodepression.

## Figures and Tables

**Table 1:** Selected characteristics extracted from the included studies (n = 37) on the immunogenicity of recombinant zoster vaccine (RZV) in adults, across sub-cohorts (n = 84).

								Immunogenicity Endpoints									
Author and Year	Location	Study Design	Immunocompromised Population	Included Individuals with History of HZ	Included Individuals Previously Vaccinated with ZVL	Co-Administration with Other Vaccines	Time between RZV2 and Blood Sampling	HI	CMI	Avidity	GMC	N	Age/Age Range	Proportion of Women	HI-VRR Responder	CMI-VRR Responders	Meta-Analysis
Laing et al., 2023	United States	RCT	-	-	-	-	One month	-	Yes	-	-	16	50–85	50.0	-	NR	-
Naficy et al., 2023 (a)	United States	RCT	-	-	NR	-	One month	Yes	-	-	Yes	100	50–59	54.8	97.0	-	Yes
Naficy et al., 2023 (b)	United States	RCT	-	-	NR	-	One month	Yes	-	-	Yes	75	60–69	54.8	94.7	-	Yes
Naficy et al., 2023 (c)	United States	RCT	-	-	NR	-	One month	Yes	-	-	Yes	53	≥70	54.8	100	-	Yes
Naficy et al., 2023 (d)	United States	RCT	-	-	NR	COVID-19 mRNA-1273	One month	Yes	-	-	Yes	102	50–59	58.1	98.0	-	Yes
Naficy et al., 2023 (e)	United States	RCT	-	-	NR	COVID-19 mRNA-1273	One month	Yes	-	-	Yes	80	60–69	58.1	97.5	-	Yes
Naficy et al., 2023 (f)	United States	RCT	-	-	NR	COVID-19 mRNA-1273	One month	Yes	-	-	Yes	48	70–88	58.1	95.8	-	Yes
Weinberg et al., 2023	United States	RCT	-	-	Yes	-	One month	Yes	-	Yes	-	80	≥50	53.0	NR	-	-
Boutry et al., 2022	Multi-country	RCT	-	-	-	-	72 months	Yes	Yes	-	-	813	≥50	60.8	NR	NR	-
Johnson et al., 2022 (a)	United States	RCT	-	-	-	-	One month	-	Yes	-	-	160	50–85	52.0	-	93.7	-
Johnson et al., 2022 (b)	United States	RCT	-	-	-	-	60 months	-	Yes	-	-	160	50–85	52.0	-	74.0	-
Min et al., 2022 (a)	Multi-country	RCT	-	-	-	-	One month	Yes	-	-	Yes	163	50–59	61.9	99.4		Yes
Min et al., 2022 (b)	Multi-country	RCT	-	-	-	-	One month	Yes	-	-	Yes	167	60–69	61.9	100		Yes
Min et al., 2022 (c)	Multi-country	RCT	-	-	-	-	One month	Yes	-	-	Yes	106	≥70	61.9	97.2		Yes
Min et al., 2022 (d)	Multi-country	RCT	-	-	-	PCV13	One month	Yes	-	-	Yes	161	50–59	57.8	99.4		Yes
Min et al., 2022 (e)	Multi-country	RCT	-	-	-	PCV13	One month	Yes	-	-	Yes	162	60–69	57.8	99.4		Yes
Min et al., 2022 (f)	Multi-country	RCT	-	-	-	PCV13	One month	Yes	-	-	Yes	104	≥70	57.8	98.1		Yes
Muchtar et al., 2022 (a)	United States	Cohort	Chronic lymphocytic leukemia and monoclonal B cell lymphocytosis (treatment naïve)	NR	Yes	-	One month	Yes	Yes	-	-	37	32–85	37.8	51.0	72.7	Only for HI
Muchtar et al., 2022 (b)	United States	Cohort	Chronic lymphocytic leukemia and monoclonal B cell lymphocytosis (BTKi treated)	NR	Yes	-	One month	Yes	Yes	-	-	25	48–82	32.0	36.0	31.6	Only for HI
Muchtar et al., 2022 (a + b)	United States	Cohort	Chronic lymphocytic leukemia and monoclonal B cell lymphocytosis	NR	Yes	-	12 months	Yes	Yes	-	-	47	32–85	35.0	34.0	NR	Only for HI
Pleyer et al., 2022 (a)	United States	RCT	Chronic lymphocytic leukemia patients (treatment naïve)	-	-	-	3 months	Yes	Yes	-	-	56	≥50	41.1	76.8	70.0	-
Pleyer et al., 2022 (b)	United States	RCT	Chronic lymphocytic leukemia patients receiving Bruton tyrosine kinase inhibitors (BTKi treated)	-	-	-	3 months	Yes	Yes	-	-	50	≥50	38.0	40.0	41.3	-
Pleyer et al., 2022 (a + b)	United States	RCT	Chronic lymphocytic leukemia patients	-	-	-	12 months	Yes	-	-	-	26	≥50	38.0	42.3	-	-
Strezova et al., 2022	Multi-country	RCT	-	-	-	-	120 months	Yes	Yes	-	-	813	≥50	60.7	NR	NR	-
Dagnew et al., 2021 (a)	Multi-country	qRCT	-	-	-	-	12 months	Yes	Yes	-	-	199	≥65	50.8	NR	NR	-
Dagnew et al., 2021 (b)	Multi-country	qRCT	-	-	Yes	-	12 months	Yes	Yes	-	-	198	≥65	51.0	NR	NR	-
Hastie et al., 2021	Multi-country	RCT	-	-	-	-	120 months	Yes	Yes	-	-	68	≥60	61.8	NR	NR	-
Hirzel et al., 2021	Canada	Cohort	Lung transplant recipients	-	NR	-	3–6 weeks	Yes	Yes	Yes	-	43	≥18	40.8	NR	NR	-
L’Huillier et al., 2021	Canada	Cohort	Solid organ transplant recipients	-	-	-	One month	Yes	Yes	Yes	-	20	≥18	52.2	55.0	NR	-
Schmid et al., 2021	United States	RCT	-	-	Yes	-	One month	-	-	Yes	-	80	50–85	NR	-	-	-
Stadtmauer et al., 2021 (a)	Multi-country	RCT	Hematopoietic stem cell transplant recipients	-	-	-	One month	Yes	Yes	-	-	26	18–49	35.4	57.7	100	Yes
Stadtmauer et al., 2021 (b)	Multi-country	RCT	Hematopoietic stem cell transplant recipients	-	-	-	One month	Yes	Yes	-	-	56	≥50	35.4	71.4	89.3	Yes
Stadtmauer et al., 2021 (c)	Multi-country	RCT	Hematopoietic stem cell transplant recipients	-	-	-	12 months	Yes	Yes	-	-	18	18–49	35.4	33.3	90.0	Yes
Stadtmauer et al., 2021 (d)	Multi-country	RCT	Hematopoietic stem cell transplant recipients	-	-	-	12 months	Yes	Yes	-	-	34	≥50	35.4	44.1	58.8	Yes
Stadtmauer et al., 2021 (e)	Multi-country	RCT	Hematopoietic stem cell transplant recipients	-	-	-	24 months	Yes	Yes	-	-	13	18–49	35.4	23.1	100	Yes
Stadtmauer et al., 2021 (f)	Multi-country	RCT	Hematopoietic stem cell transplant recipients	-	-	-	24 months	Yes	Yes	-	-	25	≥50	35.4	56.0	58.8	Yes
Zent et al., 2021	United States	Cohort	Patients with chronic lymphocytic leukemia or lymphoplasmacytic lymphoma BTKi treated	Yes	Yes	-	One month	Yes	Yes	-	-	32	≥50	34.4	75.0	78.1	-
Vink et al., 2020 (a)	Multi-country	RCT	Renal transplant recipients	-	-	-	One month	Yes	Yes	-	-	46	18–49	28.8	84.8	63.6	Yes
Vink et al., 2020 (b)	Multi-country	RCT	Renal transplant recipients	-	-	-	One month	Yes	Yes	-	-	75	≥50	28.8	77.3	76.5	Yes
Vink et al., 2020 (c)	Multi-country	RCT	Renal transplant recipients	-	-	-	6 months	Yes	Yes	-	-	41	18–49	28.8	80.5	NR	Yes
Vink et al., 2020 (d)	Multi-country	RCT	Renal transplant recipients	-	-	-	12 months	Yes	Yes	-	-	41	18–49	28.8	70.7	58.3	Yes
Bastidas et al., 2019 (a)	Multi-country	RCT	Autologous hemopoietic stem cell transplantation recipients	-	-	-	One month	Yes	Yes	-	-	82	18–78	37.1	67.0	93.0	Yes
Bastidas et al., 2019 (b)	Multi-country	RCT	Autologous hemopoietic stem cell transplantation recipients	-	-	-	12 months	Yes	-	-	-	52	18–78	37.1	41.0	-	Yes
Bastidas et al., 2019 (c)	Multi-country	RCT	Autologous hemopoietic stem cell transplantation recipients	-	-	-	24 months	Yes	-	-	-	38	18–78	37.1	45.0	-	Yes
Dagnew et al., 2019 (a)	Multi-country	RCT	Patients with hematological malignancies excluding NHBCL&CLL	-	-	-	One month	Yes	Yes	-	-	148	≥18	40.3	80.4	83.7	Yes
Dagnew et al., 2019 (b)	Multi-country	RCT	Patients with all hematological malignancies	-	-	-	One month	Yes	Yes	-	-	69	≥18	40.3	33.3		Yes
Dagnew et al., 2019 (c)	Multi-country	RCT	Patients with hematological malignancies (all)	-	-	-	12 months	Yes	Yes	-	-	165	≥18	40.3	52.1	66.7	Yes
Marechal et al., 2019 (a)	Multi-country	RCT	-	-	-	-	One month	Yes	-	-	Yes	402	≥50	58.2	98.3	-	Yes
Marechal et al., 2019 (b)	Multi-country	RCT	-	-	-	PPSV23	One month	Yes	-	-	Yes	401	≥50	61.1	98.3	-	Yes
Strezova et al., 2019 (a)	United States	RCT	-	-	-	-	One month	Yes	-	-	Yes	378	≥50	53.8	97.9	-	Yes
Strezova et al., 2019 (b)	United States	RCT	-	-	-	Tdap	One month	Yes	-	-	Yes	369	≥50	53.9	97.8	-	Yes
Vink et al., 2019 (a)	Multi-country	RCT	Patients with solid tumors before chemotherapy	-	-	-	One month	Yes	Yes	-	-	65	≥18	59.8	93.8	50.0	Yes
Vink et al., 2019 (b)	Multi-country	RCT	Patients with solid tumors during chemotherapy	-	-	-	One month	Yes	Yes	-	-	22	≥18	59.8	NR	NR	-
Cunningham et al., 2018 (a)	Multi-country	RCT	-	-	-	-	One month	Yes	Yes	-	-	1455	≥50	58.5	97.8	93.3	Yes
Cunningham et al., 2018 (b)	Multi-country	RCT	-	-	-	-	12 months	-	Yes	-	-	1384	≥50	58.5	-	57.2	Yes
Cunningham et al., 2018 (c)	Multi-country	RCT	-	-	-	-	24 months	-	Yes	-	-	1338	≥50	58.5	-	57.2	Yes
Cunningham et al., 2018 (d)	Multi-country	RCT	-	-	-	-	36 months	Yes	-	-	-	1279	≥50	58.5	77.1	NR	Yes
Lal et al., 2018 (a)	Multi-country	RCT	-	-	-	-	One month	Yes	-	-	Yes	118	≥50	75.6	96.6	-	Yes
Lal et al., 2018 (b)	Multi-country	RCT	-	-	-	-	One month	Yes	-	-	Yes	114	≥50	64.7	96.5	-	-
Lal et al., 2018 (c)	Multi-country	RCT	-	-	-	-	One month	Yes	-	-	Yes	111	≥50	68.1	94.5	-	-
Schwarz et al., 2018	Multi-country	RCT	-	-	-	-	108 months	Yes	Yes	-	-	70	≥60	61.4	NR	NR	-
Weinberg et al., 2018	United States	RCT	-	-	Yes	-	One month	-	Yes	-	-	158	≥50	54.0	-	NR	-
Godeaux et al., 2017 (a)	United States	RCT	-	Yes	NR	-	One month	Yes	-	-	Yes	31	50–59	75.0	87.1	-	Yes
Godeaux et al., 2017 (b)	United States	RCT	-	Yes	NR	-	One month	Yes	-	-	Yes	31	60–69	59.4	93.5	-	Yes
Godeaux et al., 2017 (c)	United States	RCT	-	Yes	NR	-	One month	Yes	-	-	Yes	29	≥70	62.5	86.2	-	Yes
Grupping et al., 2017 (a)	United States	qRCT	-	-	-	-	One month	Yes	Yes	-	Yes	204	≥65	51.6	NR	NR	-
Grupping et al., 2017 (b)	United States	qRCT	-	-	Yes	-	One month	Yes	Yes	-	Yes	204	≥65	50.7	NR	NR	-
Schwarz et al., 2017 (a)	Multi-country	RCT	-	-	-	-	One month	Yes	-	-	Yes	388	≥50	52.5	97.9	-	Yes
Schwarz et al., 2017 (b)	Multi-country	RCT	-	-	-	IIV4	One month	Yes	-	-	Yes	382	≥50	51.1	95.8	-	Yes
Strezova et al., 2017 (a)	Multi-country	RCT	-	-	-	-	One month	Yes	-	-	-	210	50–89	53.2	95.7	-	Yes
Strezova et al., 2017 (b)	Multi-country	RCT	-	-	-	-	One month	Yes	-	-	-	210	50–91	59.9	97.6	-	Yes
Strezova et al., 2017 (c)	Multi-country	RCT	-	-	-	-	One month	Yes	-	-	-	202	50–91	52.8	97.5	-	Yes
Vink et al., 2017 (a)	Japan	RCT	-	-	-	-	One month	Yes	-	-	Yes	29	≥50	50.0	100	-	-
Vink et al., 2017 (b)	Japan	RCT	-	-	-	-	One month	Yes	-	-	Yes	29	≥50	50.0	100	-	-
Vink et al., 2017 (c)	Japan	RCT	-	-	-	-	12 months	Yes	-	-	Yes	28	≥50	50.0	89.3	-	Yes
Vink et al., 2017 (d)	Japan	RCT	-	-	-	-	12 months	Yes	-	-	Yes	30	≥50	50.0	83.3	-	Yes
Chlibek et al., 2016	Multi-country	RCT	-	-	-	-	72 months	Yes	Yes	-	-	129	60–84	60.5	NR	NR	-
Berkowitz et al., 2015	Multi-country	RCT	People living with HIV	-	-	-	One month	Yes	Yes	-	-	53		6.8	98.1	85.7	Yes
Chlibek et al., 2014	Multi-country	RCT	-	-	-	-	One month	Yes	Yes	-	-	166	60–84	60.5	NR	NR	-
Stadtmauer et al., 2014 (a)	United States	RCT	Autologous hemopoietic stem cell transplantation recipients	-	-	-	One month	Yes	Yes	-	-	27	42–68	32.3	76.9	75.0	Only for CMI
Stadtmauer et al., 2014 (b)	United States	RCT	Autologous hemopoietic stem cell transplantation recipients	-	-	-	12 months	Yes	Yes	-	-	23	42–68	32.3	54.5	NR	-
Chlibek et al., 2013	Multi-country	RCT	-	-	-	-	One month	Yes	Yes	-	-	148	≥50	54.0	NR	NR	-
Leroux-Roels et al., 2012 (a)	Belgium	RCT	-	-	-	-	One month	Yes	Yes	-	-	10	18–30	50.0	100	NR	-
Leroux-Roels et al., 2012 (b)	Belgium	RCT	-	-	-	-	One month	Yes	Yes	-	-	45	50–70	73.0	100	NR	-

Notes: The number of participants in each report is reported as the highest number and may not be the same for all outcomes. The proportion of women, unless specifically reported for each subgroup, is the one described for the entire sample. Letters in brackets in the first column indicate the different reports extracted from individual studies included. For the complete list of included studies, please refer to the Supplementary Materials, Supplementary S1. Abbreviations: HZ, herpes zoster; ZVL, zoster live vaccine; RVZ2, dose 2 of the recombinant zoster vaccine; HI, humoral immunity; CMI, cell-mediated immunity; VRR, vaccine response rate; GMC, geometric mean concentration of antibody; RCT, randomized controlled trial; qRCT, quasi-randomized control trial; NR, not reported; PCV13, 13-valent pneumococcal conjugate vaccine; PPSV23, 23-valent pneumococcal polysaccharide vaccine; Tdap, tetanus–diphtheria–pertussis vaccine; IIV4, quadrivalent seasonal inactivated influenza vaccine; HIV, human immunodeficiency virus.

## Data Availability

All data are available in the manuscript.

## Supplementary Materials

The following supporting information can be downloaded at: https://www.mdpi.com/article/10.3390/vaccines12050527/s1, Table S1: Search strategy; Table S2: PICOS framework; Table S3. Full texts not included in the systematic review, with reasons for exclusion; Table S4: Definition of endpoints not accepted for inclusion in the meta-analysis for the vaccine response rate (VRR); Table S5: Pooled estimates of vaccine response rate for humoral immunity one month after RZV-dose 2 according to selected subgroups; Table S6: Random-effects meta-regression results of the association between vaccine response rate for humoral immunity (one month after RZV-dose 2) and mediators; Table S7: Pooled estimates of vaccine response rate for cell-mediated immunity one month after RZV-dose 2 according to selected subgroups; Table S8: Random-effects meta-regression results of the association between vaccine response rate for cell-mediated immunity (one month after RZV-dose 2) and mediators; Table S9: Risk-of-bias (RoB) of randomized controlled trials; Table S10: Risk-of-bias (RoB) of non-randomized studies (cohort studies); Table S11: Egger’s regression asymmetry test for random-effects meta-analyses; Figure S1: Median CD4^2+^ T-cell frequencies by time between the two RZV doses; Figure S2: Funnel plot of random-effects meta-analysis of humoral VRR one month after RZV-dose 2; Figure S3: Funnel plot of random-effects meta-analysis of CMI-specific VRR one month after RZV-dose 2.

## References

[1] Cohen J.I. (2013). Herpes Zoster. N. Engl. J. Med..

[2] Sampathkumar P., Drage L.A., Martin D.P. (2009). Herpes Zoster (Shingles) and Postherpetic Neuralgia. Mayo Clin. Proc..

[3] (2023). Centers for Disease Control and Prevention—National Center for Immunization and Respiratory Diseases, Division of Viral Diseases. Shingles (Herpes Zoster). https://www.cdc.gov/shingles/hcp/clinical-overview.html.

[4] Thomas S.L., Hall A.J. (2004). What Does Epidemiology Tell Us about Risk Factors for Herpes Zoster?. Lancet Infect. Dis..

[5] Dagnew A.F., Vink P., Drame M., Willer D.O., Salaun B., Schuind A.E. (2021). Immune Responses to the Adjuvanted Recombinant Zoster Vaccine in Immunocompromised Adults: A Comprehensive Overview. Hum. Vaccines Immunother..

[6] Weinberg A., Levin M.J., Abendroth A., Arvin A.M., Moffat J.F. (2010). VZV T Cell-Mediated Immunity. Varicella-Zoster Virus.

[7] Sullivan N.L., Reuter-Monslow M.A., Sei J., Durr E., Davis C.W., Chang C., McCausland M., Wieland A., Krah D., Rouphael N. (2018). Breadth and Functionality of Varicella-Zoster Virus Glycoprotein-Specific Antibodies Identified after Zostavax Vaccination in Humans. J. Virol..

[8] Park S.Y., Levin M.J., Canniff J., Johnson M., Schmid D.S., Weinberg A. (2022). Development of Antibody-Dependent Cellular Cytotoxicity in Response to Recombinant and Live-Attenuated Herpes Zoster Vaccines. Npj Vaccines.

[9] Ito M., Ihara T., Grose C., Starr S. (1985). Human Leukocytes Kill Varicella-Zoster Virus-Infected Fibroblasts in the Presence of Murine Monoclonal Antibodies to Virus-Specific Glycoproteins. J. Virol..

[10] Harbecke R., Cohen J.I., Oxman M.N. (2021). Herpes Zoster Vaccines. J. Infect. Dis..

[11] Malavige G.N., Jones L., Black A.P., Ogg G.S. (2008). Varicella Zoster Virus Glycoprotein E-Specific CD4+ T Cells Show Evidence of Recent Activation and Effector Differentiation, Consistent with Frequent Exposure to Replicative Cycle Antigens in Healthy Immune Donors. Clin. Exp. Immunol..

[12] Didierlaurent A.M., Collignon C., Bourguignon P., Wouters S., Fierens K., Fochesato M., Dendouga N., Langlet C., Malissen B., Lambrecht B.N. (2014). Enhancement of Adaptive Immunity by the Human Vaccine Adjuvant AS01 Depends on Activated Dendritic Cells. J. Immunol..

[13] Vandepapelière P., Horsmans Y., Moris P., Van Mechelen M., Janssens M., Koutsoukos M., Van Belle P., Clement F., Hanon E., Wettendorff M. (2008). Vaccine Adjuvant Systems Containing Monophosphoryl Lipid A and QS21 Induce Strong and Persistent Humoral and T Cell Responses against Hepatitis B Surface Antigen in Healthy Adult Volunteers. Vaccine.

[14] Lal H., Cunningham A.L., Godeaux O., Chlibek R., Diez-Domingo J., Hwang S.-J., Levin M.J., McElhaney J.E., Poder A., Puig-Barberà J. (2015). Efficacy of an Adjuvanted Herpes Zoster Subunit Vaccine in Older Adults. N. Engl. J. Med..

[15] Cunningham A.L., Lal H., Kovac M., Chlibek R., Hwang S.-J., Díez-Domingo J., Godeaux O., Levin M.J., McElhaney J.E., Puig-Barberà J. (2016). Efficacy of the Herpes Zoster Subunit Vaccine in Adults 70 Years of Age or Older. N. Engl. J. Med..

[16] Curran D., Kim J.H., Matthews S., Dessart C., Levin M.J., Oostvogels L., Riley M.E., Schmader K.E., Cunningham A.L., McNeil S.A. (2021). Recombinant Zoster Vaccine Is Efficacious and Safe in Frail Individuals. J. Am. Geriatr. Soc..

[17] Strezova A., Diez-Domingo J., Al Shawafi K., Tinoco J.C., Shi M., Pirrotta P., Mwakingwe-Omari A., Adams M., Ahonen A., Zoster-049 Study Group (2022). Long-Term Protection Against Herpes Zoster by the Adjuvanted Recombinant Zoster Vaccine: Interim Efficacy, Immunogenicity, and Safety Results up to 10 Years After Initial Vaccination. Open Forum Infect. Dis..

[18] Cunningham A.L., Heineman T.C., Lal H., Godeaux O., Chlibek R., Hwang S.-J., McElhaney J.E., Vesikari T., Andrews C., Choi W.S. (2018). Immune Responses to a Recombinant Glycoprotein E Herpes Zoster Vaccine in Adults Aged 50 Years or Older. J. Infect. Dis..

[19] Stadtmauer E.A., Sullivan K.M., El Idrissi M., Salaun B., Alonso Alonso A., Andreadis C., Anttila V.-J., Bloor A.J., Broady R., Cellini C. (2021). Adjuvanted Recombinant Zoster Vaccine in Adult Autologous Stem Cell Transplant Recipients: Polyfunctional Immune Responses and Lessons for Clinical Practice. Hum. Vaccines Immunother..

[20] Weinberg A., Scott Schmid D., Leung J., Johnson M.J., Miao C., Levin M.J. (2023). Predictors of 5-Year Persistence of Antibody Responses to Zoster Vaccines. J. Infect. Dis..

[21] Schwarz T.F., Volpe S., Catteau G., Chlibek R., David M.P., Richardus J.H., Lal H., Oostvogels L., Pauksens K., Ravault S. (2018). Persistence of Immune Response to an Adjuvanted Varicella-Zoster Virus Subunit Vaccine for up to Year Nine in Older Adults. Hum. Vaccines Immunother..

[22] Bastidas A., De La Serna J., El Idrissi M., Oostvogels L., Quittet P., López-Jiménez J., Vural F., Pohlreich D., Zuckerman T., Issa N.C. (2019). Effect of Recombinant Zoster Vaccine on Incidence of Herpes Zoster after Autologous Stem Cell Transplantation: A Randomized Clinical Trial. JAMA.

[23] Pleyer C., Laing K.J., Ali M.A., McClurkan C.L., Soto S., Ahn I.E., Nierman P., Maddux E., Lotter J., Superata J. (2022). BTK Inhibitors Impair Humoral and Cellular Responses to Recombinant Zoster Vaccine in CLL. Blood Adv..

[24] Hirzel C., L’Huillier A.G., Ferreira V.H., Marinelli T., Ku T., Ierullo M., Miao C., Schmid D.S., Juvet S., Humar A. (2021). Safety and Immunogenicity of Adjuvanted Recombinant Subunit Herpes Zoster Vaccine in Lung Transplant Recipients. Am. J. Transplant..

[25] Page M.J., McKenzie J.E., Bossuyt P.M., Boutron I., Hoffmann T.C., Mulrow C.D., Shamseer L., Tetzlaff J.M., Akl E.A., Brennan S.E. (2021). The PRISMA 2020 Statement: An Updated Guideline for Reporting Systematic Reviews. BMJ.

[26] PROSPERO Immunogenicity of Recombinant Zoster Vaccine: A Systematic Review, Meta-Analysis and Meta-Regression [CRD42023459621]. https://www.crd.york.ac.uk/prospero/display_record.php?RecordID=459621.

[27] Sterne J.A.C., Savović J., Page M.J., Elbers R.G., Blencowe N.S., Boutron I., Cates C.J., Cheng H.-Y., Corbett M.S., Eldridge S.M. (2019). RoB 2: A Revised Tool for Assessing Risk of Bias in Randomised Trials. BMJ.

[28] Joanna Briggs Institute Critical Appraisal Tools for Use in JBI Systematic Reviews: Checklist for Non-Randomized Experimental Studies. https://joannabriggs.org/critical-appraisal-tools.

[29] Deeks J., Higgins J., Altman D. (2023). Analysing Data and Undertaking Meta-Analyses. Cochrane Handbook for Systematic Reviews of Interventions.

[30] Schmid D.S., Miao C., Leung J., Johnson M., Weinberg A., Levin M.J. (2021). Comparative Antibody Responses to the Live-Attenuated and Recombinant Herpes Zoster Vaccines. J. Virol..

[31] Moris P., Van Der Most R., Leroux-Roels I., Clement F., Dramé M., Hanon E., Leroux-Roels G.G., Van Mechelen M. (2011). H5N1 Influenza Vaccine Formulated with AS03A Induces Strong Cross-Reactive and Polyfunctional CD4 T-Cell Responses. J. Clin. Immunol..

[32] Wang N. (2023). How to Conduct a Meta-Analysis of Proportions in R: A Comprehensive Tutorial. J. Behav. Data. Sci..

[33] Egger M., Smith G.D., Schneider M., Minder C. (1997). Bias in Meta-Analysis Detected by a Simple, Graphical Test. BMJ.

[34] Schwarzer G., Carpenter J.R., Rücker G. (2015). Meta-Analysis with R.

[35] Harrer M., Cuijpers P., Furukawa T.A., Ebert D.D. (2022). Doing Meta-Analysis with R: A Hands-on Guide.

[36] R Foundation for Statistical Computing. www.R-project.org.

[37] Muchtar E., Koehler A.B., Johnson M.J., Rabe K.G., Ding W., Call T.G., Leis J.F., Kenderian S.S., Hayman S.R., Wang Y. (2022). Humoral and Cellular Immune Responses to Recombinant Herpes Zoster Vaccine in Patients with Chronic Lymphocytic Leukemia and Monoclonal B Cell Lymphocytosis. Am. J. Hematol..

[38] Zent C.S., Brady M.T., Delage C., Strawderman M., Laniewski N., Contant P.N., Kanagaiah P., Sangster M.Y., Barr P.M., Chu C.C. (2021). Short Term Results of Vaccination with Adjuvanted Recombinant Varicella Zoster Glycoprotein E during Initial BTK Inhibitor Therapy for CLL or Lymphoplasmacytic Lymphoma. Leukemia.

[39] Chlibek R., Bayas J.M., Collins H., De La Pinta M.L.R., Ledent E., Mols J.F., Heineman T.C. (2013). Safety and Immunogenicity of an AS01-Adjuvanted Varicella-Zoster Virus Subunit Candidate Vaccine Against Herpes Zoster in Adults >=50 Years of Age. J. Infect. Dis..

[40] Godeaux O., Kovac M., Shu D., Grupping K., Campora L., Douha M., Heineman T.C., Lal H. (2017). Immunogenicity and Safety of an Adjuvanted Herpes Zoster Subunit Candidate Vaccine in Adults ≥ 50 Years of Age with a Prior History of Herpes Zoster: A Phase III, Non-Randomized, Open-Label Clinical Trial. Hum. Vaccines Immunother..

[41] Naficy A., Kuxhausen A., Pirrotta P., Leav B., Miller J., Anteyi K., Danier J., Breuer T., Mwakingwe-Omari A. (2023). No Immunological Interference or Safety Concerns When Adjuvanted Recombinant Zoster Vaccine Is Coadministered with a Coronavirus Disease 2019 mRNA-1273 Booster Vaccine in Adults Aged 50 Years and Older: A Randomized Trial. Clin. Infect. Dis..

[42] Min J.-Y., Mwakingwe-Omari A., Riley M., Molo L.Y., Soni J., Girard G., Danier J. (2022). The Adjuvanted Recombinant Zoster Vaccine Co-Administered with the 13-Valent Pneumococcal Conjugate Vaccine in Adults Aged ≥ 50 Years: A Randomized Trial. J. Infect..

[43] Maréchal C., Lal H., Poder A., Ferguson M., Enweonye I., Heineman T.C., Hervé C., Rheault P., Talli J., Wauters D. (2018). Immunogenicity and Safety of the Adjuvanted Recombinant Zoster Vaccine Co-Administered with the 23-Valent Pneumococcal Polysaccharide Vaccine in Adults ≥50 Years of Age: A Randomized Trial. Vaccine.

[44] Strezova A., Lal H., Enweonye I., Campora L., Beukelaers P., Segall N., Heineman T.C., Schuind A.E., Oostvogels L. (2019). The Adjuvanted Recombinant Zoster Vaccine Co-Administered with a Tetanus, Diphtheria and Pertussis Vaccine in Adults Aged ≥ 50 Years: A Randomized Trial. Vaccine.

[45] Schwarz T.F., Aggarwal N., Moeckesch B., Schenkenberger I., Claeys C., Douha M., Godeaux O., Grupping K., Heineman T.C., Fauqued M.L. (2017). Immunogenicity and Safety of an Adjuvanted Herpes Zoster Subunit Vaccine Coadministered with Seasonal Influenza Vaccine in Adults Aged 50 Years or Older. J. Infect. Dis..

[46] Lal H., Poder A., Campora L., Geeraerts B., Oostvogels L., Vanden Abeele C., Heineman T.C. (2018). Immunogenicity, Reactogenicity and Safety of 2 Doses of an Adjuvanted Herpes Zoster Subunit Vaccine Administered 2, 6 or 12 Months Apart in Older Adults: Results of a Phase III, Randomized, Open-Label, Multicenter Study. Vaccine.

[47] Grupping K., Campora L., Douha M., Heineman T.C., Klein N.P., Lal H., Peterson J., Vastiau I., Oostvogels L. (2017). Immunogenicity and Safety of the HZ/Su Adjuvanted Herpes Zoster Subunit Vaccine in Adults Previously Vaccinated with a Live Attenuated Herpes Zoster Vaccine. J. Infect. Dis..

[48] Vink P., Shiramoto M., Ogawa M., Eda M., Douha M., Heineman T., Lal H. (2017). Safety and Immunogenicity of a Herpes Zoster Subunit Vaccine in Japanese Population Aged ≥ 50 Years When Administered Subcutaneously vs. Intramuscularly. Hum. Vaccines Immunother..

[49] L’Huillier A.G., Hirzel C., Ferreira V.H., Ierullo M., Ku T., Selzner N., Schiff J., Juvet S., Miao C., Schmid D.S. (2021). Evaluation of Recombinant Herpes Zoster Vaccine for Primary Immunization of Varicella-Seronegative Transplant Recipients. Transplantation.

[50] Johnson M.J., Liu C., Ghosh D., Lang N., Levin M.J., Weinberg A. (2022). Cell-Mediated Immune Responses After Administration of the Live or the Recombinant Zoster Vaccine: 5-Year Persistence. J. Infect. Dis..

[51] Weinberg A., Kroehl M.E., Johnson M.J., Hammes A., Reinhold D., Lang N., Levin M.J. (2018). Comparative Immune Responses to Licensed Herpes Zoster Vaccines. J. Infect. Dis..

[52] Vink P., Delgado Mingorance I., Maximiano Alonso C., Rubio-Viqueira B., Jung K.H., Rodriguez Moreno J.F., Grande E., Marrupe Gonzalez D., Lowndes S., Puente J. (2019). Immunogenicity and Safety of the Adjuvanted Recombinant Zoster Vaccine in Patients with Solid Tumors, Vaccinated before or during Chemotherapy: A Randomized Trial. Cancer.

[53] Vink P., Ramon Torrell J.M., Sanchez Fructuoso A., Kim S.-J., Kim S., Zaltzman J., Ortiz F., Campistol Plana J.M., Fernandez Rodriguez A.M., Rebollo Rodrigo H. Immunogenicity and Safety of the Adjuvanted Recombinant Zoster Vaccine in Chronically Immunosuppressed Adults Following Renal Transplant: A Phase III, Randomized Clinical Trial. Clin. Infect. Dis..

[54] Leroux-Roels I., Leroux-Roels G., Clement F., Vandepapelière P., Vassilev V., Ledent E., Heineman T.C. (2012). A Phase 1/2 Clinical Trial Evaluating Safety and Immunogenicity of a Varicella Zoster Glycoprotein E Subunit Vaccine Candidate in Young and Older Adults. J. Infect. Dis..

[55] Dagnew A.F., Klein N.P., Hervé C., Kalema G., Di Paolo E., Peterson J., Salaun B., Schuind A. (2021). The Adjuvanted Recombinant Zoster Vaccine in Adults Aged ≥ 65 Years Previously Vaccinated with a Live-Attenuated Herpes Zoster Vaccine. J. Infect. Dis..

[56] Laing K.J., Ford E.S., Johnson M.J., Levin M.J., Koelle D.M., Weinberg A. (2023). Recruitment of Naïve CD4+ T Cells by the Recombinant Zoster Vaccine Correlates with Persistent Immunity. J. Clin. Investig..

[57] Hastie A., Catteau G., Enemuo A., Mrkvan T., Salaun B., Volpe S., Smetana J., Rombo L., Schwarz T., Pauksens K. (2021). Immunogenicity of the Adjuvanted Recombinant Zoster Vaccine: Persistence and Anamnestic Response to Additional Doses Administered 10 Years After Primary Vaccination. J. Infect. Dis..

[58] Chlibek R., Smetana J., Pauksens K., Rombo L., Van Den Hoek J.A.R., Richardus J.H., Plassmann G., Schwarz T.F., Ledent E., Heineman T.C. (2014). Safety and Immunogenicity of Three Different Formulations of an Adjuvanted Varicella-Zoster Virus Subunit Candidate Vaccine in Older Adults: A Phase II, Randomized, Controlled Study. Vaccine.

[59] Chlibek R., Pauksens K., Rombo L., Van Rijckevorsel G., Richardus J.H., Plassmann G., Schwarz T.F., Catteau G., Lal H., Heineman T.C. (2016). Long-Term Immunogenicity and Safety of an Investigational Herpes Zoster Subunit Vaccine in Older Adults. Vaccine.

[60] Boutry C., Hastie A., Diez-Domingo J., Tinoco J.C., Yu C.-J., Andrews C., Beytout J., Caso C., Cheng H.-S., Cheong H.J. (2022). The Adjuvanted Recombinant Zoster Vaccine Confers Long-Term Protection against Herpes Zoster: Interim Results of an Extension Study of the Pivotal Phase 3 Clinical Trials ZOE-50 and ZOE-70. Clin. Infect. Dis..

[61] Dagnew A.F., Ilhan O., Lee W.-S., Woszczyk D., Kwak J.-Y., Bowcock S., Sohn S.K., Rodriguez Macías G., Chiou T.-J., Quiel D. (2019). Immunogenicity and Safety of the Adjuvanted Recombinant Zoster Vaccine in Adults with Haematological Malignancies: A Phase 3, Randomised, Clinical Trial and Post-Hoc Efficacy Analysis. Lancet Infect. Dis..

[62] Stadtmauer E.A., Sullivan K.M., Marty F.M., Dadwal S.S., Papanicolaou G.A., Shea T.C., Mossad S.B., Andreadis C., Young J.-A.H., Buadi F.K. (2014). A Phase 1/2 Study of an Adjuvanted Varicella-Zoster Virus Subunit Vaccine in Autologous Hematopoietic Cell Transplant Recipients. Blood.

[63] Herishanu Y., Avivi I., Aharon A., Shefer G., Levi S., Bronstein Y., Morales M., Ziv T., Shorer Arbel Y., Scarfò L. (2021). Efficacy of the BNT162b2 mRNA COVID-19 Vaccine in Patients with Chronic Lymphocytic Leukemia. Blood.

[64] Yri O.E., Torfoss D., Hungnes O., Tierens A., Waalen K., Nordøy T., Dudman S., Kilander A., Wader K.F., Østenstad B. (2011). Rituximab Blocks Protective Serologic Response to Influenza A (H1N1) 2009 Vaccination in Lymphoma Patients during or within 6 Months after Treatment. Blood.

[65] Bedognetti D., Zoppoli G., Massucco C., Zanardi E., Zupo S., Bruzzone A., Sertoli M.R., Balleari E., Racchi O., Messina M. (2011). Impaired Response to Influenza Vaccine Associated with Persistent Memory B Cell Depletion in Non-Hodgkin’s Lymphoma Patients Treated with Rituximab-Containing Regimens. J. Immunol..

[66] Vlachonikola E., Stamatopoulos K., Chatzidimitriou A. (2021). T Cells in Chronic Lymphocytic Leukemia: A Two-Edged Sword. Front. Immunol..

[67] Hirzel C., Kumar D. (2018). Influenza Vaccine Strategies for Solid Organ Transplant Recipients. Curr. Opin. Infect. Dis..

[68] Gourishankar S., McDermid J.C., Jhangri G.S., Preiksaitis J.K. (2004). Herpes zoster infection following solid organ transplantation: Incidence, risk factors and outcomes in the current immunosuppressive era. Am. J. Transplant..

[69] US Centers for Disease Control and Prevention—Advisory Committee on Immunization Practices (2023). General Best Practice Guidelines for Immunization—Timing and Spacing of Immunobiologics. https://www.cdc.gov/vaccines/hcp/acip-recs/general-recs/timing.html.

[70] Alexander K.E., Tong P.L., Macartney K., Beresford R., Sheppeard V., Gupta M. (2018). Live Zoster Vaccination in an Immunocompromised Patient Leading to Death Secondary to Disseminated Varicella Zoster Virus Infection. Vaccine.

[71] Whitmire J.K. (2011). Induction and Function of Virus-Specific CD4+ T Cell Responses. Virology.

[72] Levin M.J., Kroehl M.E., Johnson M.J., Hammes A., Reinhold D., Lang N., Weinberg A. (2018). Th1 Memory Differentiates Recombinant from Live Herpes Zoster Vaccines. J. Clin. Investig..

[73] Leroux-Roels G., Van Belle P., Vandepapeliere P., Horsmans Y., Janssens M., Carletti I., Garçon N., Wettendorff M., Van Mechelen M. (2015). Vaccine Adjuvant Systems Containing Monophosphoryl Lipid A and QS-21 Induce Strong Humoral and Cellular Immune Responses against Hepatitis B Surface Antigen Which Persist for at Least 4 Years after Vaccination. Vaccine.

[74] Leroux-Roels G., Marchant A., Levy J., Van Damme P., Schwarz T.F., Horsmans Y., Jilg W., Kremsner P.G., Haelterman E., Clément F. (2016). Impact of Adjuvants on CD4^+^ T Cell and B Cell Responses to a Protein Antigen Vaccine: Results from a Phase II, Randomized, Multicenter Trial. Clin. Immunol..

[75] Coccia M., Collignon C., Hervé C., Chalon A., Welsby I., Detienne S., Van Helden M.J., Dutta S., Genito C.J., Waters N.C. (2017). Cellular and Molecular Synergy in AS01-Adjuvanted Vaccines Results in an Early IFNγ Response Promoting Vaccine Immunogenicity. Npj Vaccines.

[76] Qi Q., Cavanagh M.M., Le Saux S., Wagar L.E., Mackey S., Hu J., Maecker H., Swan G.E., Davis M.M., Dekker C.L. (2016). Defective T Memory Cell Differentiation after Varicella Zoster Vaccination in Older Individuals. PLOS Pathog..

[77] Tseng H.F., Harpaz R., Luo Y., Hales C.M., Sy L.S., Tartof S.Y., Bialek S., Hechter R.C., Jacobsen S.J. (2016). Declining Effectiveness of Herpes Zoster Vaccine in Adults Aged ≥ 60 Years. J. Infect. Dis..

[78] Tafuri S., Bianchi F.P., Stefanizzi P. (2022). The Public Health and the Question of the “Best Vaccine”. Vaccine.

[79] Baker W.L., Michael White C., Cappelleri J.C., Kluger J., Coleman C.I., From the Health Outcomes, Policy, and Economics (HOPE) Collaborative Group (2009). Understanding Heterogeneity in Meta-Analysis: The Role of Meta-Regression. Int. J. Clin. Pract..

[80] Berkowitz E.M., Moyle G., Stellbrink H.-J., Schürmann D., Kegg S., Stoll M., El Idrissi M., Oostvogels L., Heineman T.C., for the Zoster-015 HZ/su Study Group (2015). Safety and Immunogenicity of an Adjuvanted Herpes Zoster Subunit Candidate Vaccine in HIV-Infected Adults: A Phase 1/2a Randomized, Placebo-Controlled Study. J. Infect. Dis..

