# Supplementary material for: Immunogenicity of Recombinant Zoster Vaccine: A Systematic Review, Meta-Analysis, and Meta-Regression
Source: Vaccines (Basel). 2024 May 11;12(5):527. doi: 10.3390/vaccines12050527 (PMC11125663; doi:10.3390/vaccines12050527)
Supplement: Supplementary file 1 [file vaccines-12-00527-s001.zip › vaccines-2950164-supplementary.pdf]

vaccines MDPI

Supplementary material

Supplementary S1

List of studies that met the inclusion criteria and were included in the systematic review (N = 37)

1. Laing KJ, Ford ES, Johnson MJ, Levin MJ, Koelle DM, Weinberg A. Recruitment of naïve CD4+ T cells by the recombinant zoster vaccine correlates with persistent immunity. J Clin Invest. Published online October 3, 2023. doi:10.1172/JCI172634
2. Naficy A, Kuxhausen A, Pirrotta P, et al. No Immunological Interference or Safety Concerns When Adjuvanted Recombinant Zoster Vaccine Is Coadministered With a Coronavirus Disease 2019 mRNA-1273 Booster Vaccine in Adults Aged 50 Years and Older: A Randomized Trial. Clin Infect Dis. Published online June 19, 2023:ciad361. doi:10.1093/cid/ciad361
3. Weinberg A, Scott Schmid D, Leung J, Johnson MJ, Miao C, Levin MJ. Predictors of 5-Year Persistence of Antibody Responses to Zoster Vaccines. J Infect Dis. Published online May 4, 2023:jiad132. doi:10.1093/infdis/jiad132
4. Boutry C, Hastie A, Diez-Domingo J, et al. The Adjuvanted Recombinant Zoster Vaccine Confers Long-Term Protection Against Herpes Zoster: Interim Results of an Extension Study of the Pivotal Phase 3 Clinical Trials ZOE-50 and ZOE-70. Clin Infect Dis. 2022;74(8):1459-1467. doi:10.1093/cid/ciab629
5. Johnson MJ, Liu C, Ghosh D, Lang N, Levin MJ, Weinberg A. Cell-Mediated Immune Responses After Administration of the Live or the Recombinant Zoster Vaccine: 5-Year Persistence. J Infect Dis. 2022;225(8):1477-1481. doi:10.1093/infdis/jiab580
6. Min JY, Mwakingwe-Omari A, Riley M, et al. The adjuvanted recombinant zoster vaccine co-administered with the 13-valent pneumococcal conjugate vaccine in adults aged ≥50 years: A randomized trial. J Infect. 2022;84(4):490-498. doi:10.1016/j.jinf.2021.12.033
7. Muchtar E, Koehler AB, Johnson MJ, et al. Humoral and cellular immune responses to recombinant herpes zoster vaccine in patients with chronic lymphocytic leukemia and monoclonal B cell lymphocytosis. Am J Hematol. 2022;97(1):90-98. doi:10.1002/ajh.26388
8. Pleyer C, Laing KJ, Ali MA, et al. BTK inhibitors impair humoral and cellular responses to recombinant zoster vaccine in CLL. Blood Adv. 2022;6(6):1732-1740. doi:10.1182/bloodadvances.2021006574
9. Strezova A, Diez-Domingo J, Al Shawafi K, Tinoco JC, Shi M, Pirrotta P, Mwakingwe-Omari A; Zoster-049 Study Group. Long-term Protection Against Herpes Zoster by the Adjuvanted Recombinant Zoster Vaccine: Interim Efficacy, Immunogenicity, and Safety Results up to 10 Years After Initial Vaccination. Open Forum Infect Dis. 2022;9(10):ofac485. doi: 10.1093/ofid/ofac485.
10. Dagnew AF, Klein NP, Hervé C, et al. The Adjuvanted Recombinant Zoster Vaccine in Adults Aged ≥65 Years Previously Vaccinated With a Live-Attenuated Herpes Zoster Vaccine. J Infect Dis. 2021;224(7):1139-1146. doi:10.1093/infdis/jiaa083
11. Hastie A, Catteau G, Enemuo A, et al. Immunogenicity of the Adjuvanted Recombinant Zoster Vaccine: Persistence and Anamnestic Response to Additional Doses Administered 10 Years After Primary Vaccination. J Infect Dis. 2021;224(12):2025-2034. doi:10.1093/infdis/jiaa300
12. Hirzel C, L'Huillier AG, Ferreira VH, et al. Safety and immunogenicity of adjuvanted recombinant subunit herpes zoster vaccine in lung transplant recipients. Am J Transplant. 2021;21(6):2246-2253. doi:10.1111/ajt.16534
13. L'Huillier AG, Hirzel C, Ferreira VH, et al. Evaluation of Recombinant Herpes Zoster Vaccine for Primary Immunization of Varicella-seronegative Transplant Recipients. Transplantation. 2021;105(10):2316-2323. doi:10.1097/TP.0000000000003621
14. Schmid DS, Miao C, Leung J, Johnson M, Weinberg A, Levin MJ. Comparative Antibody Responses to the Live-Attenuated and Recombinant Herpes Zoster Vaccines. Shisler JL, ed. J Virol. 2021;95(12):e00240-21. doi:10.1128/JVI.00240-21
15. Stadtmauer EA, Sullivan KM, El Idrissi M, et al. Adjuvanted recombinant zoster vaccine in adult autologous stem cell transplant recipients: polyfunctional immune responses and lessons for clinical practice. Hum Vaccines Immunother. 2021;17(11):4144-4154. doi:10.1080/21645515.2021.1953346

16. Zent CS, Brady MT, Delage C, et al. Short term results of vaccination with adjuvanted recombinant varicella zoster glycoprotein E during initial BTK inhibitor therapy for CLL or lymphoplasmacytic lymphoma. Leukemia. 2021;35(6):1788-1791. doi:10.1038/s41375-020-01074-4
17. Vink P, Ramon Torrell JM, Sanchez Fructuoso A, et al. Immunogenicity and Safety of the Adjuvanted Recombinant Zoster Vaccine in Chronically Immunosuppressed Adults Following Renal Transplant: a Phase III, Randomized Clinical Trial. Clin Infect Dis. Published online March 7, 2019. doi:10.1093/cid/ciz177
18. Bastidas A, De La Serna J, El Idrissi M, et al. Effect of Recombinant Zoster Vaccine on Incidence of Herpes Zoster After Autologous Stem Cell Transplantation: A Randomized Clinical Trial. JAMA. 2019;322(2):123. doi:10.1001/jama.2019.9053
19. Dagnew AF, Ilhan O, Lee WS, et al. Immunogenicity and safety of the adjuvanted recombinant zoster vaccine in adults with haematological malignancies: a phase 3, randomised, clinical trial and post-hoc efficacy analysis. Lancet Infect Dis. 2019;19(9):988-1000. doi:10.1016/S1473-3099(19)30163-X
20. Maréchal C, Lal H, Poder A, et al. Immunogenicity and safety of the adjuvanted recombinant zoster vaccine co-administered with the 23-valent pneumococcal polysaccharide vaccine in adults ≥50 years of age: A randomized trial. Vaccine. 2018;36(29):4278-4286. doi:10.1016/j.vaccine.2018.05.110
21. Strezova A, Lal H, Enweonye I, et al. The adjuvanted recombinant zoster vaccine co-administered with a tetanus, diphtheria and pertussis vaccine in adults aged ≥50 years: A randomized trial. Vaccine. 2019;37(39):5877-5885. doi:10.1016/j.vaccine.2019.08.001
22. Vink P, Delgado Mingorance I, Maximiano Alonso C, et al. Immunogenicity and safety of the adjuvanted recombinant zoster vaccine in patients with solid tumors, vaccinated before or during chemotherapy: A randomized trial. Cancer. 2019;125(8):1301-1312. doi:10.1002/cncr.31909
23. Cunningham AL, Heineman TC, Lal H, et al. Immune Responses to a Recombinant Glycoprotein E Herpes Zoster Vaccine in Adults Aged 50 Years or Older. J Infect Dis. 2018;217(11):1750-1760. doi:10.1093/infdis/jiy095
24. Lal H, Poder A, Campora L, et al. Immunogenicity, reactogenicity and safety of 2 doses of an adjuvanted herpes zoster subunit vaccine administered 2, 6 or 12 months apart in older adults: Results of a phase III, randomized, open-label, multicenter study. Vaccine. 2018;36(1):148-154. doi:10.1016/j.vaccine.2017.11.019
25. Schwarz TF, Volpe S, Catteau G, et al. Persistence of immune response to an adjuvanted varicella-zoster virus subunit vaccine for up to year nine in older adults. Hum Vaccines Immunother. 2018;14(6):1370-1377. doi:10.1080/21645515.2018.1442162
26. Weinberg A, Kroehl ME, Johnson MJ, et al. Comparative Immune Responses to Licensed Herpes Zoster Vaccines. J Infect Dis. 2018;218(suppl_2):S81-S87. doi:10.1093/infdis/jiy383
27. Godeaux O, Kovac M, Shu D, et al. Immunogenicity and safety of an adjuvanted herpes zoster subunit candidate vaccine in adults ≥ 50 years of age with a prior history of herpes zoster: A phase III, non-randomized, open-label clinical trial. Hum Vaccines Immunother. 2017;13(5):1051-1058. doi:10.1080/21645515.2016.1265715
28. Grupping K, Campora L, Douha M, et al. Immunogenicity and Safety of the HZ/su Adjuvanted Herpes Zoster Subunit Vaccine in Adults Previously Vaccinated With a Live Attenuated Herpes Zoster Vaccine. J Infect Dis. 2017;216(11):1343-1351. doi:10.1093/infdis/jix482
29. Schwarz TF, Aggarwal N, Moeckesch B, et al. Immunogenicity and Safety of an Adjuvanted Herpes Zoster Subunit Vaccine Coadministered With Seasonal Influenza Vaccine in Adults Aged 50 Years or Older. J Infect Dis. 2017;216(11):1352-1361. doi:10.1093/infdis/jix481
30. Strezova A, Godeaux O, Aggarwal N, Leroux-Roels G, Lopez-Fauqued M, Van Damme P, et al. A randomized lot-to-lot immunogenicity consistency study of the candidate zoster vaccine HZ/su. Vaccine. 2017;35(48 Pt B):6700-6706. doi: 10.1016/j.vaccine.2017.10.017.
31. Vink P, Shiramoto M, Ogawa M, et al. Safety and immunogenicity of a Herpes Zoster subunit vaccine in Japanese population aged ≥50 years when administered subcutaneously vs. intramuscularly. Hum Vaccines Immunother. 2017;13(3):574-578. doi:10.1080/21645515.2016.1232787
32. Chlibek R, Pauksens K, Rombo L, et al. Long-term immunogenicity and safety of an investigational herpes zoster subunit vaccine in older adults. Vaccine. 2016;34(6):863-868. doi:10.1016/j.vaccine.2015.09.073
33. Berkowitz EM, Moyle G, Stellbrink HJ, et al. Safety and Immunogenicity of an Adjuvanted Herpes Zoster Subunit Candidate Vaccine in HIV-Infected Adults: A Phase 1/2a Randomized, Placebo-Controlled Study. J Infect Dis. 2015;211(8):1279-1287.
34. Chlibek R, Smetana J, Pauksens K, et al. Safety and immunogenicity of three different formulations of an adjuvanted varicella-zoster virus subunit candidate vaccine in older adults: A phase II, randomized, controlled study. Vaccine. 2014;32(15):1745-1753. doi:10.1016/j.vaccine.2014.01.019

35. Stadtmauer EA, Sullivan KM, Marty FM, et al. A phase 1/2 study of an adjuvanted varicella-zoster virus subunit vaccine in autologous hematopoietic cell transplant recipients. Blood. 2014;124(19):2921-2929. doi:10.1182/blood-2014-04-573048
36. Chlibek R, Bayas JM, Collins H, et al. Safety and Immunogenicity of an AS01-adjuvanted Varicella-zoster Virus Subunit Candidate Vaccine Against Herpes Zoster in Adults >=50 Years of Age. J Infect Dis. 2013;208(12):1953-1961. doi:10.1093/infdis/jit365
37. Leroux-Roels I, Leroux-Roels G, Clement F, et al. A Phase 1/2 Clinical Trial Evaluating Safety and Immunogenicity of a Varicella Zoster Glycoprotein E Subunit Vaccine Candidate in Young and Older Adults. J Infect Dis. 2012;206(8):1280-1290. doi:10.1093/infdis/jis497

Supplementary S2

Supplementary tables

Table S1. Search strategy

Database	Search strategy	Reports retrieved
MEDLINE/PubMed	("Herpes Zoster Vaccine"[Mesh] OR ("Herpes Zoster/prevention and control"[Mesh] AND "Vaccination"[Mesh]) OR (zoster vaccine*[TIAB]) OR (zoster immunization[TIAB]) OR (zoster immunisation[TIAB]) OR (VZV vaccin*[TIAB])) AND ("Antibodies, Viral"[Mesh:NoExp] OR "Serologic Tests"[Mesh] OR seroconversion[TIAB] OR seroconverted[TIAB] OR serological[TIAB] OR antibod*[TIAB] OR immunogenicity[TIAB] OR immunization[TIAB] OR immunisation[TIAB] OR (immune response[TIAB]) OR immunity[TIAB] OR effectiveness[TIAB] OR efficacy[TIAB]) NOT (Animals [Mesh] NOT Humans [Mesh])	1125
Embase	('varicella zoster vaccine':ti,ab,kw OR (('herpes zoster' NEXT/2 (vaccin* OR immunization OR immunisation)):ab,ti)) AND ('zoster antibody' OR 'serodiagnosis'/de OR 'drug efficacy'/de OR immunogenicity:ti,ab,de OR efficacy:ti,ab,de OR effectiveness:ti,ab,de OR effectivity:ti,ab,de OR antibod*:ti,ab OR seroconversion:ti,ab OR 'response to vaccination':ti,ab OR ((response NEAR/3 'zoster vaccination'):ti,ab))	494
Web of Science	zoster vaccin* (Title) and (immunogenicity OR efficacy OR effectiveness OR antibod* OR (response to vaccination)) (All Fields)	744
WHO Global Index Medicus	herpes zoster vaccin*	182

Table S2. PICOS framework

Population	Adults aged 18 years or older.
Intervention	Two doses of Herpes zoster adjuvanted recombinant subunit vaccine (RZV), consisting of 50μg of recombinant VZV glycoprotein E and the liposome-based AS01$_B$ adjuvant system (containing 50μg of 3-O-desacyl- 4′-monophosphoryl lipid A and 50μg of *Quillaja saponaria* Molina, fraction 21 [QS21]).
Comparators	None or, where available, age group, sex, primary or secondary immunosuppression, history of herpes zoster, previous vaccination with live-attenuated zoster vaccine (ZVL), co-administration of RZV with other vaccines (including candidate vaccines).
Outcomes	Proportion of subjects who tested positive for anti-gE humoral and cell-mediated immunity (defined as vaccine response rate [VRR]), avidity index, geometric mean concentration (GMC) of antibody (related to within-study comparison).
Study design	Randomized control trials (RCTs), quasi-randomised control trials (qRCTs), cohort and case control studies, and research letter with original data.

Table S3. Full texts not included in the systematic review, with reason for exclusion

N	First author and year of publication	Title	Reason for exclusion
1	Lal et al., 2013	Safety and immunogenicity of an AS01-adjuvanted varicella zoster virus subunit candidate vaccine (HZ/su)	Data not presented in a suitable form
2	Cohen et al., 2014	Detection of Antibodies to Varicella-Zoster Virus in Recipients of the Varicella Vaccine by Using a Luciferase Immunoprecipitation System Assay	No data on immune response to RZV
3	Diez-Domingo et al., 2016	Efficacy and immunogenicity of an investigational subunit adjuvanted herpes zoster vaccine in older adults in Europe: Results from the ZOE-50 and ZOE-70 efficacy studies	Conference abstract
4	McElhaney et al., 2016	Efficacy, immunogenicity and safety of an investigational subunit adjuvanted herpes zoster vaccine in adults aged 60 years and older: Results from the zoe-50 and zoe-70 efficacy studies	Conference abstract
5	Poder et al., 2016	Immunogenicity and safety of 2 doses of an investigational herpes zoster subunit vaccine administered 2, 6 or 12 months apart in adults 50 years and older: Results of a phase III, randomized, open-label, multicenter trial	Conference abstract
6	Bao et al., 2016	Immunogenicity of varicella zoster virus glycoprotein E DNA vaccine	Non-human study
7	Verna et al., 2016	La vacuna de subunidades recombinantes para herpes zoster demostró ser eficaz en adultos mayores/ Adjuvanted herpes zoster subunit vaccine proved to be effective in older adults	Commentary
8	Weinberg et al., 2017	A comparison of the immunogenicity of a live attenuated herpes zoster vaccine (ZV) and the recombinant gE/AS01B candidate vaccine in older adults	Conference abstract
9	Pauksens et al., 2017	Persistence of immune response to an adjuvanted varicella-zoster virus subunit candidate vaccine for up to year 9 in older adults	Conference abstract
10	McElhaney et al., 2018	Efficacy, Immunogenicity and Safety of adjuvanted Herpes Zoster-Virus Subunit Vaccine Candidates in Adults from 60 Years and older: Results from the Efficacy Studies ZOE-50 and ZOE-70	Conference abstract
11	Mrkvan et al., 2018	Persistence of immune response and safety of an adjuvanted recombinant zoster vaccine in older adults previously vaccinated with a live-attenuated herpes zoster vaccine: End-of-study results of a phase III, group-matched, clinical trial	Conference abstract

12	Levin et al., 2018	Th1 memory differentiates recombinant from live herpes zoster vaccines	Data not presented in a suitable form
13	Sullivan et al., 2019	Immunogenicity and safety of an adjuvanted herpes zoster subunit vaccine in adult autologous hematopoietic stem cell transplant recipients: Phase 3, randomized, placebo-controlled, ZOEHSCT clinical trial	Conference abstract
14	Oostvogels et al., 2019	Medical conditions at enrollment do not impact efficacy and safety of the adjuvanted recombinant zoster vaccine: a pooled post-hoc analysis of two parallel randomized trials	No data on immune response to RZV
15	Righi et al., 2019	Serological and T Cell Responses After Varicella Zoster Virus Vaccination in HIV-Positive Patients Undergoing Renal Dialysis	No data on immune response to RZV
16	Yang et al., 2019	Evaluation of Varicella-zoster virus-specific cell-mediated immunity by interferon-γ Enzyme-Linked Immunosorbent Assay in adults ≥50 years of age administered a herpes zoster vaccine	No data on immune response to RZV
17	Verolet et al., 2019	Long-term Seroprotection of Varicella-zoster Immunization in Pediatric Liver Transplant Recipients	No data on immune response to RZV
18	Muchtar et al., 2020	Immunogenicity of a Recombinant Herpes Zoster Vaccine in Patients with Chronic Lymphocytic Leukemia	Conference abstract
19	Sweiss et al., 2020	High rates of varicella zoster virus antibody seroconversion and persistence after administration of the adjuvanted, recombinant varicella zoster vaccine in multiple myeloma patients undergoing active treatment	Conference abstract
20	Kallmark et al., 2020	Immunogenicity of Adjuvanted Herpes Zoster Subunit Vaccine in Rheumatoid Arthritis Patients Treated with Janus Kinase Inhibitors and Controls: Preliminary Results	Conference abstract
21	Barghash et al., 2020	Recombinant Herpes Zoster Vaccine (RZV) after Heart Transplantation: A Single Center Experience	No data on immune response to RZV
22	Camargo et al., 2020	Immunogenicity and Safety of the Adjuvanted Recombinant Zoster Vaccine in Adult Hematopoietic Cell Transplant Recipients: A Single Center Study	Conference abstract
23	Camargo et al., 2020	Reduced immunogenicity of the adjuvanted recombinant zoster vaccine after hematopoietic cell transplant: a pilot study	No data on anti-gE response
24	Curran et al., 2021	Recombinant Zoster Vaccine Is Efficacious and Safe in Frail Individuals	Same data as in other studies (Cunningham et al., 2018)
25	Pleyer et al., 2021	Effect of Bruton tyrosine kinase inhibitor on efficacy of adjuvanted recombinant hepatitis B and zoster vaccines.	Same data as in other studies (Interim analysis of Pleyer C, et al. BTK inhibitors impair humoral and

			cellular responses to recombinant zoster vaccine in CLL. Blood Adv. 2022;6(6):1732-1740)
26	Callegaro et al., 2022	Association Between Immunogenicity and Reactogenicity: A Post Hoc Analysis of 2 Phase 3 Studies with the Adjuvanted Recombinant Zoster Vaccine.	Same data as in other studies (Post hoc analysis of ZOE data)
27	Koldehoff et al., 2022	Cellular Immune Response after Vaccination with an Adjuvanted, Recombinant Zoster Vaccine in Allogeneic Hematopoietic Stem Cell Transplant Recipients	No data on anti-gE response
28	Lindemann et al., 2022	Prospective, Longitudinal Study on Specific Cellular Immune Responses after Vaccination with an Adjuvanted, Recombinant Zoster Vaccine in Kidney Transplant Recipients	No data on anti-gE response
29	Galgani et al., 2023	Immunogenicity and safety of the non-typable Haemophilus influenzae-Moraxella catarrhalis (NTHi-Mcat) vaccine administered following the recombinant zoster vaccine versus administration alone: Results from a randomized, phase 2a, non-inferiority trial	No data on immune response to RZV
30	Chen et al., 2023	Vaccination against Varicella Zoster Virus Infection in Less Developed Regions of Guangdong, China: A Cross-Sectional Serosurveillance Study	No data on immune response to RZV
31	Venerito et al., 2023	Immunogenicity and Safety of Adjuvanted Recombinant Zoster Vaccine in Rheumatoid Arthritis Patients on Anti-Cellular Biologic Agents or JAK Inhibitors: A Prospective Observational Study	No data on anti-gE response

Abbreviations: RZV, recombinant zoster vaccine.

Table S4. Definition of endpoints not accepted for inclusion in the meta-analysis for the vaccine response rate (VRR)

Author, year	Definition not accepted for seroconversion
Laing et al., 2023	CMI-specific VRR not reported.
Weinberg et al., 2023	Humoral- and CMI-specific VRR not reported.
Boutry et al., 2022	Humoral- and CMI-specific VRR not reported.
Johnson et al., 2022	Not all $CD4^+$ T cell activation markers considered.
Muchtar et al., 2022	Not all $CD4^+$ T cell activation markers considered.
Pleyer et al., 2022	Type of test for humoral VRR: Luciferase immunoprecipitation assay (LIPS). Time from vaccination to blood sample collection: 3 months.
Strezova et al., 2022	Humoral- and CMI-specific VRR not reported.
Dagnew et al., 2021	Humoral- and CMI-specific VRR not reported.
Hastie et al., 2021	Humoral- and CMI-specific VRR not reported.
Hirzel et al., 2021	Time between vaccine doses up to 6 months. Time from vaccination to blood sample collection: 3–6 weeks. Humoral- and CMI-specific VRR not reported.
L'Huillier et al., 2021	Definitions not accepted for seropositivity: Humoral immunity was expressed as mean (adjusted) in optical density rather than an antibody concentration. CMI-specific VRR not reported.
Schmid et al., 2021	Humoral- and CMI-specific VRR not calculated.
Zent et al., 2021	Time between vaccine doses up to three months.
Lal et al., 2018	Report (b) and (c) not included for time between vaccine doses of 6 and 12 months, respectively
Schwarz et al., 2018	Humoral- and CMI-specific VRR not reported.
Weinberg et al., 2018	Humoral-specific VRR not analysed. CMI-specific VRR not reported.
Grupping et al., 2017	Humoral- and CMI-specific VRR not reported.
Vink et al., 2017 (cohorts a and b)	Definitions not accepted for seropositivity: Anti-gE titre equal to or the lower detection limit of ELISA of 18 international units/mL
Chlibek et al., 2016	Definitions not accepted for seropositivity: Anti-gE titre equal to or above the detection limit of the ELISA of 18 international units/mL. Humoral- and CMI-specific VRR not reported.
Chlibek et al., 2014	Definitions not accepted for seropositivity: Anti-gE titre equal to or above the detection limit of the enzyme-linked immunosorbent assay (ELISA) of 18 international units/mL. Humoral- and CMI-specific VRR not reported.
Stadtmauer et al., 2014	Definitions not accepted for seropositivity: Anti-gE titre equal to or above the detection limit of ELISA of 18 international units/mL
Chlibek et al., 2013	Definitions not accepted for seropositivity: Anti-gE titre equal to or the lower detection limit of ELISA of 18 international units/mL. Supplementary material with VRR data for both humoral and CMI immunity not retrieved at https://academic.oup.com/jid/article/208/12/1953/2192556
Leroux-Roels et al., 2012	Definitions not accepted for seropositivity: Anti-gE titre equal to or above the detection limit of ELISA of 195 international units/mL. CMI-specific VRR not reported.

Abbreviations: VRR, vaccine response rate; CMI, cell-mediated immunity; ELISA, enzyme-linked immunosorbent assay.

Table S5. Pooled estimates of vaccine response rate for humoral immunity one month after RZV-dose 2 according to selected subgroups

Subgroup	Reports (N)	Vaccine response rate (95% Confidence Interval)	Heterogeneity	*P* value for heterogeneity between subgroups
**Overall**	37	0.9515 [0.9185; 0.9715]	94.2%	
**Age at vaccination**				< 0.0001
≥18	7	0.7987 [0.5880; 0.9169]	91.8%	
≥30	1	0.5100 [0.3533; 0.6648]	-	
≥40	1	0.3600 [0.1991; 0.5600]	-	
≥50	18	0.9690 [0.9496; 0.9810]	89.2%	
≥60	5	0.9839 [0.9487; 0.9951]	17.1%	
≥70	5	0.9692 [0.9271; 0.9874]	45.3%	
**Immunocompetent adults**				< 0.0001
Yes	23	0.9779 [0.9731; 0.9819]	0.0%	
No	14	0.7760 [0.6466; 0.8677]	87.8%	
**Co-administration with other vaccines**				0.003
No	28	0.9333 [0.8789; 0.9643]	94.8%	
Yes	9	0.9792 [0.9682; 0.9865]	22.8%	
**Dosing interval**				< 0.0001
One month	3	0.7559 [0.3812; 0.9396]	96.5%	
One/two months	3	0.7577 [0.6651; 0.8312]	61.4%	
Two months	31	0.9656 [0.9412; 0.9800]	91.0%	
**Including subjects with history of herpes zoster**				0.01
No	32	0.9623 [0.9360; 0.9780]	94.0%	
Yes	3	0.8899 [0.8075; 0.9397]	0.0%	
**Study design**				< 0.0001
Randomised controlled trial	34	0.9589 [0.9330; 0.9750]	93.5%	
Cohort	2	0.4495 [0.3311; 0.5739]	25.6%	
**Multi-country**				0.62
No	13	0.9420 [0.8685; 0.9756]	91.9%	
Yes	24	0.9558 [0.9149; 0.9775]	95.3%	

Abbreviations: RZV, recombinant zoster vaccine.

Table S6. Random-effects meta-regression results of the association between vaccine response rate for humoral immunity (one month after RZV-dose 2) and mediators

	Univariable		Multivariable ^	
**Mediator**	**Coefficient (95% CI)**	**P value**	**Coefficient (95% CI)**	**P value**
**Age at vaccination**				
≥18	Reference	-	Reference	-
≥30	-1.2910 (-3.5179; 0.9359)	0.2559	-1.6734 (-3.3314; -0.0155)	**0.0479**
≥40	-1.9284 (-4.2146; 0.3579)	0.0983	-2.2644 (-3.9918; -0.5370)	**0.0102**
≥50	2.1112 (1.1621; 3.0602)	<.0001	-0.1709 (-1.3210; 0.9791)	0.7708
≥60	2.7785 (1.3830; 4.1740)	<.0001	0.4258 (-1.0225; 1.8741)	0.5645
≥70	2.1959 (0.8292; 3.5627)	0.0016	-0.0648 (-1.5125; 1.3829)	0.9301
**Sex (increasing proportion of female vaccinees)**	0.0678 (0.0350; 0.1007)	<.0001	0.0071 (-0.0215; 0.0356)	0.6287
**Immunocompetent adults**				
No	Reference	-	Reference	-
Yes	2.7464 (2.1465; 3.3463)	<.0001	2.1134 (1.2359; 2.9908)	**<.0001**
**Co-administration with other vaccines**				
No	Reference	-	Reference	-
Yes	1.4826 (0.2726; 2.6925)	0.0163	0.0431 (-0.6640; 0.7502)	0.9049
**Dosing interval**				
One month	Reference	-	Reference	-
One/two months	0.1058 (-2.1588; 2.3704)	0.9270	0.3312 (-0.9472; 1.6096)	0.6116
Two months	2.1914 (0.5025; 3.8803)	0.0110	0.7421 (-0.5242; 2.0084)	0.2507
**Including subjects with history of Herpes Zoster**				
No	Reference	-		
Yes	-0.9869 (-2.8183; 0.8446)	0.2909		
**Study design**				
Cohort	Reference	-		
Randomised controlled trial	3.4150 (1.3578; 5.4722)	0.0011		
**Multi-country**				
Yes	Reference	-		
No (United States)	-0.2687 (-1.4268; 0.8894)	0.6493		

^ Redundant predictors dropped from the multivariable model.
Abbreviations: RZV, recombinant zoster vaccine; 95%CI, 95% confidence interval.

Table S7. Pooled estimates of vaccine response rate for cell-mediated immunity one month after RZV-dose 2 according to selected subgroups

Subgroup	Reports (N)	Vaccine response rate (95% Confidence Interval)	Heterogeneity	*P* value for heterogeneity between subgroups
**Overall**	10	0.8459 [0.7520; 0.9086]	71.9%	
**Age at vaccination**				0.20
≥18	6	0.8352 [0.6702; 0.9267]	70.7%	
≥40	1	0.7500 [0.5436; 0.8831]	-	
≥50	3	0.9040 [0.7992; 0.9571]	59.4%	
**Immunocompetent adults**				0.02
No	9	0.8252 [0.7216; 0.8958]	58.2%	
Yes	1	0.9330 [0.8799; 0.9636]	-	
**Co-administration with other vaccines**				Not estimable
No	10	0.8459 [0.7520; 0.9086]	71.9%	
Yes	0	-	-	
**Dosing interval**				0.13
One month	2	0.7014 [0.4247; 0.8820]	86.9%	
One/two months	3	0.8251 [0.6358; 0.9273]	65.4%	
Two months	5	0.8957 [0.8252; 0.9398]	47.4%	
**Including subjects with history of herpes zoster**				
No	10	0.8459 [0.7520; 0.9086]	71.9%	
Yes	0	-	-	
**Study design**				Not estimable
Randomised controlled trial	10	0.8459 [0.7520; 0.9086]	71.9%	
Cohort	0	-	-	
**Multi-country**				0.24
No	1	0.7500 [0.5436; 0.8831]	-	
Yes	9	0.8550 [0.7536; 0.9192]	74.0%	

Abbreviations: RZV, recombinant zoster vaccine.

Table S8. Random-effects meta-regression results of the association between vaccine response rate for cell-mediated immunity (one month after RZV-dose 2) and mediators

	Univariable		Multivariable	
**Mediator**	**Coefficient (95% CI)**	**P value**	**Coefficient (95% CI)**	**P value**
**Age at vaccination**				
≥18	Reference	-		
≥40	-0.4319 (-2.2124; 1.3487)	0.6345		
≥50	0.5547 (-0.6575; 1.7669)	0.3698		
**Sex (increasing proportion of female vaccinees)**	-0.0045 (-0.0431; 0.0341)	0.8186		
**Immunocompetent adults**				
No	Reference	-		
Yes	1.1322 (0.3617; 2.6261)	0.1374		
**Dosing interval**				
One month	Reference	-		
One/two months	0.6969 (-0.6680; 2.0619)	0.3170		
Two months	1.3005 (0.0882; 2.5127)	**0.0355**		
**Multi-country**				
Yes	Reference	-		
No (United States)	-0.6288 (-2.4952; 1.2377)	0.5091		

Abbreviations: RZV, recombinant zoster vaccine; 95%CI, 95% confidence interval.

Table S9. Risk of bias (RoB) of randomized controlled trials

Study, year of publication	Domain 1	Domain 2	Domain 3	Domain 4	Domain 5	Overall RoB
Laing, 2023	Low	Low	Low	Low	Low	**Low**
Naficy, 2023	Low	Low	Low	Low	Low	**Low**
Weinberg, 2023	Low	Low	Low	Low	Low	**Low**
Boutry, 2022	Low	Low	Low	Low	Low	**Low**
Johnson, 2022	Low	Low	Low	Low	Low	**Low**
Min, 2022	Some concerns	Low	Low	Low	Low	**Some Concerns**
Pleyer, 2022	Low	Low	Low	Low	Low	**Low**
Strezova, 2022	High	Low	Low	Low	Low	**High**
Dagnew, 2021	Low	Low	Low	Low	Low	**Low**
Hastie, 2021	High	Low	Low	Low	Low	**High**
Schmid, 2021	Low	Low	Low	Low	Low	**Low**
Stadtmauer, 2021	Low	Low	Low	Low	Low	**Low**
Vink, 2020	Low	Low	Low	Low	Low	**Low**
Bastidas, 2019	Low	Low	Low	Low	Low	**Low**
Dagnew, 2019	Low	Low	High	Low	Low	**High**
Marechal, 2019	Low	Low	Low	Low	Low	**Low**
Strezova, 2019	Low	Low	Low	Low	Low	**Low**
Vink, 2019	Low	Low	Low	Low	Low	**Low**
Cunningham, 2018	Low	Low	Low	Low	Low	**Low**
Lal, 2018	Low	Low	Low	Low	Low	**Low**
Schwarz, 2018	Low	Low	Low	Low	Low	**Low**
Weinberg, 2018	Low	Low	Low	Low	Low	**Low**
Godeaux, 2017	High	Low	Low	Low	Low	**High**
Grupping, 2017	High	Low	Low	Low	Low	**High**
Schwarz, 2017	Low	Low	Low	Low	Low	**Low**

Study, year of publication	Domain 1	Domain 2	Domain 3	Domain 4	Domain 5	Overall RoB
Strezova, 2017	Low	Low	Low	Low	Low	**Low**
Vink, 2017	Some Concerns	Low	Low	Low	Low	**Some Concerns**
Chlibek, 2016	Low	Low	Low	Low	Low	**Low**
Berkowitz, 2015	Low	Low	Low	Low	Low	**Low**
Chlibek, 2014	Low	Low	Low	Low	Low	**Low**
Stadtmauer, 2014	Low	Low	Low	Low	Low	**Low**
Chlibek, 2013	Low	Low	Low	Low	Low	**Low**
Leroux-Roels, 2012	Low	Low	Low	Low	Low	**Low**

RoB items included: domain 1 (randomization), domain. 2 (deviation from intended intervention), domain 3 (outcome missingness), domain 4 (outcome measurement), domain 5 (selective reporting).
Each domain was graded through a set of response options – from "Low" to "Some Concerns" to "High", or Not/Applicable (NA).

RoB of randomised controlled trials has been evaluated using the Cochrane risk of bias tool (see: Sterne JAC, Savović J, Page MJ et al. RoB 2: a revised tool for assessing risk of bias in randomised trials. BMJ. 2019;366:l4898).

Table S10. Risk of bias (RoB) of non-randomized studies (cohort studies)

Study, year of publication	Domain 1	Domain 2	Domain 3	Domain 4	Domain 5	Domain 6	Domain 7	Domain 8	Domain 9	Overall RoB
Muchtar, 2022	Yes	Yes	No	Yes	Yes	Yes	Yes	Yes	Yes	**Low**
Hirzel, 2021	Yes	Yes	Yes	No	Yes	Yes	Yes	Yes	Yes	**High**
L'Huillier, 2021	Yes	Yes	Yes	No	Yes	Yes	Yes	Yes	Yes	**High**
Zent, 2021	Yes	Yes	Yes	Yes	Yes	Yes	Yes	Yes	Yes	**Low**

RoB items included: domain 1 (clarity of 'cause' and 'effect'), domain 2 (comparison similarities), domain 3 (comparisons of similar treatments), domain 4 (control group), domain 5 (multiple measurements pre and post), domain 6 (follow up), domain 7 (similarity of outcome measures), domain 8 (measurement reliability), domain 9 (appropriate statistical analysis).

Each domain was graded through a set of response options: "Yes", "No", "Unclear" or Not/Applicable (NA).

RoB of non-randomised studies was evaluated using the Joanna Briggs Institute checklist for cohort studies (see: Moola S, Munn Z, Tufanaru C, et al. Chapter 7: Systematic reviews of etiology and risk. In: Aromataris E, Munn Z (Editors). JBI Manual for Evidence Synthesis. JBI, 2020. Available from https://synthesismanual.jbi.global).

Table S11. Egger's regression asymmetry test for random-effects meta-analyses

Model	Intercept (95%CI)	t	*P* value
Humoral VRR one month after RZV-dose 2	2.82 (-0.13; 5.76)	1.874	0.07
CMI-specific VRR one month after RZV-dose 2	-0.21 (-4.51; 4.09)	-0.096	0.93

Abbreviations: RZV, recombinant zoster vaccine; VRR, vaccine response rate; CMI, cell-mediated immunity; 95%CI, 95% confidence interval.

Supplementary S3

Supplementary figures

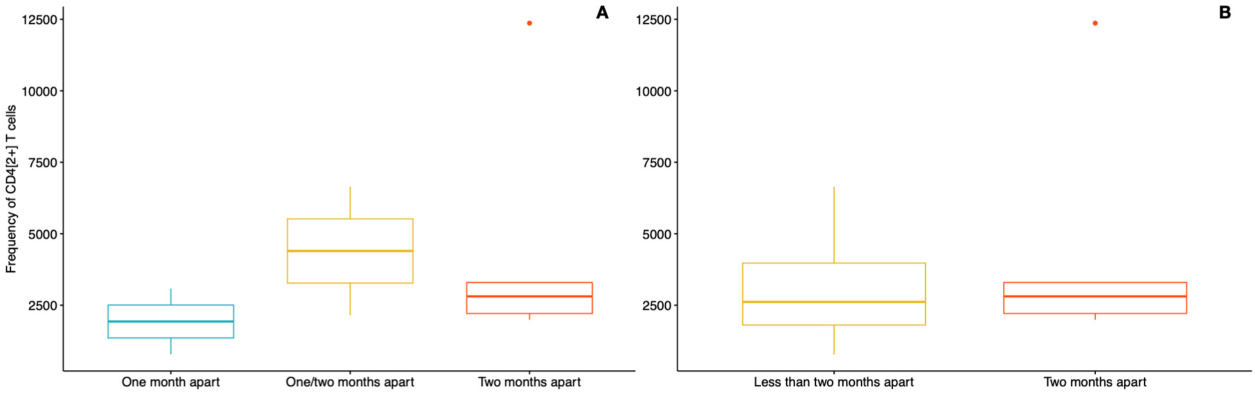

Figure S1. Median CD4^{2+} T-cell frequencies by time between the two doses of recombinant zoster vaccine (RZV)
Median CD4^{2+} T-cell frequencies are sourced from reports included in the meta-analysis of vaccine response rates of cell-mediated immunity. Panel A presents the results based on dosing interval as included in the meta-analysis; Panel B includes a sensitivity analysis by combining all studies that administered doses within two months. Kruskal Wallis test was used to determine whether the medians of the groups were unequal.

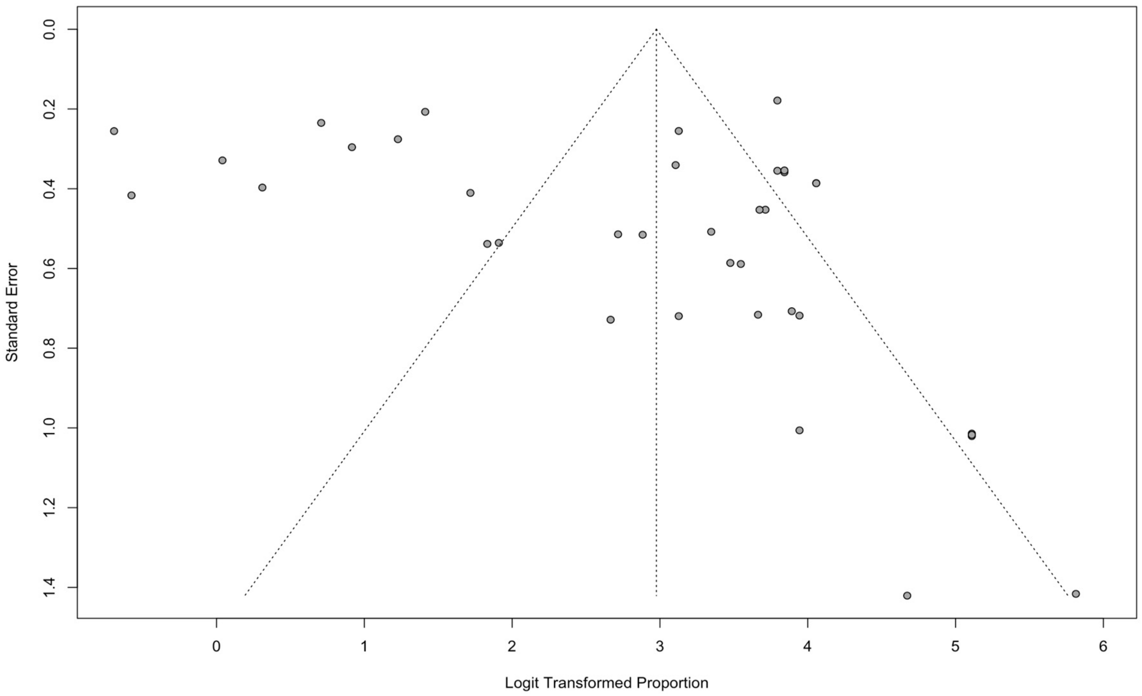

Figure S2. Funnel plot of random-effects meta-analysis of humoral VRR one month after RZV dose 2
Abbreviations: VRR, vaccine response rate; RZV, recombinant zoster vaccine.

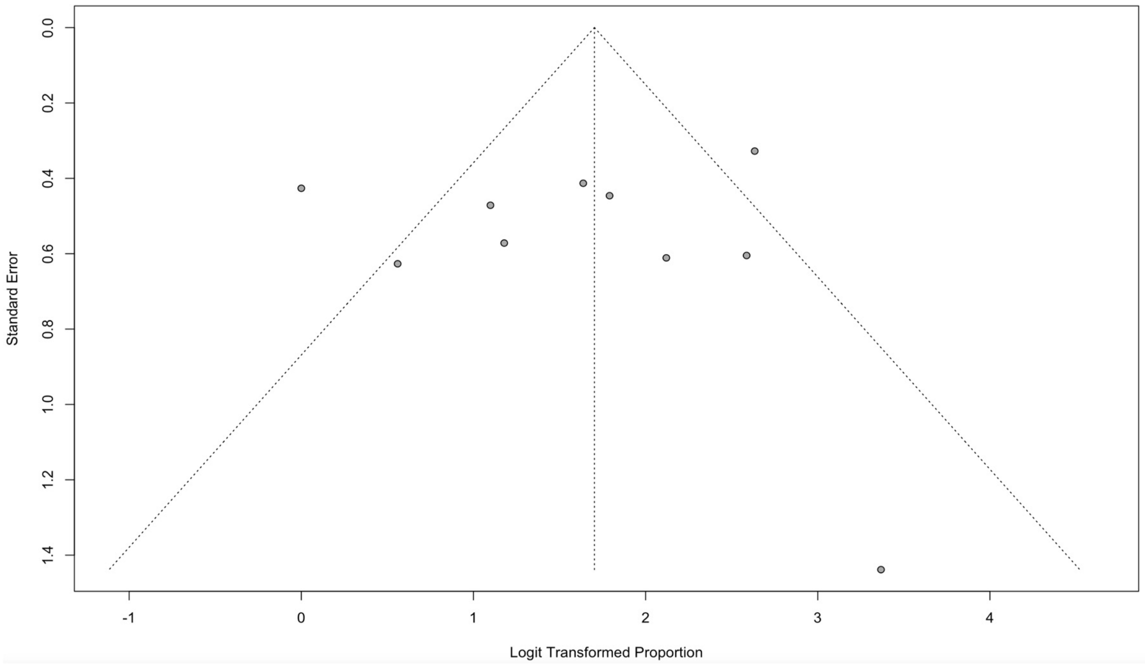

Figure S3. Funnel plot of random-effects meta-analysis of CMI-specific VRR one month after RZV dose 2
Abbreviations: VRR, vaccine response rate; RZV, recombinant zoster vaccine; CMI, cell-mediated immunity.

Disclaimer/Publisher's Note: The statements, opinions and data contained in all publications are solely those of the individual author(s) and contributor(s) and not of MDPI and/or the editor(s). MDPI and/or the editor(s) disclaim responsibility for any injury to people or property resulting from any ideas, methods, instructions or products referred to in the content.
